# Improved Elucidation of Biological Processes Linked to Diabetic Nephropathy by Single Probe-Based Microarray Data Analysis

**DOI:** 10.1371/journal.pone.0002937

**Published:** 2008-08-13

**Authors:** Clemens D. Cohen, Maja T. Lindenmeyer, Felix Eichinger, Alexander Hahn, Martin Seifert, Anton G. Moll, Holger Schmid, Eva Kiss, Elisabeth Gröne, Hermann-Josef Gröne, Matthias Kretzler, Thomas Werner, Peter J. Nelson

**Affiliations:** 1 Nephrology Clinic and Institute of Physiology with Center of Integrative Human Physiology, University Hospital and University, Zurich, Switzerland; 2 Clinical Biochemistry Group, Medical Policlinic, University of Munich, Munich, Germany; 3 Division of Nephrology, Department of Medicine, University of Michigan, Ann Arbor, Michigan, United States of America; 4 Genomatix Software GmbH, Munich, Germany; 5 Cellular and Molecular Pathology, German Cancer Research Center, Heidelberg, Germany; L' Istituto di Biomedicina ed Immunologia Molecolare, Consiglio Nazionale delle Ricerche, Italy

## Abstract

**Background:**

Diabetic nephropathy (DN) is a complex and chronic metabolic disease that evolves into a progressive fibrosing renal disorder. Effective transcriptomic profiling of slowly evolving disease processes such as DN can be problematic. The changes that occur are often subtle and can escape detection by conventional oligonucleotide DNA array analyses.

**Methodology/Principal Findings:**

We examined microdissected human renal tissue with or without DN using Affymetrix oligonucleotide microarrays (HG-U133A) by standard Robust Multi-array Analysis (RMA). Subsequent gene ontology analysis by Database for Annotation, Visualization and Integrated Discovery (DAVID) showed limited detection of biological processes previously identified as central mechanisms in the development of DN (e.g. inflammation and angiogenesis). This apparent lack of sensitivity may be associated with the gene-oriented averaging of oligonucleotide probe signals, as this includes signals from cross-hybridizing probes and gene annotation that is based on out of date genomic data. We then examined the same CEL file data using a different methodology to determine how well it could correlate transcriptomic data with observed biology. ChipInspector (CI) is based on single probe analysis and *de novo* gene annotation that bypasses probe set definitions. Both methods, RMA and CI, used at default settings yielded comparable numbers of differentially regulated genes. However, when verified by RT-PCR, the single probe based analysis demonstrated reduced background noise with enhanced sensitivity and fewer false positives.

**Conclusions/Significance:**

Using a single probe based analysis approach with *de novo* gene annotation allowed an improved representation of the biological processes linked to the development and progression of DN. The improved analysis was exemplified by the detection of Wnt signaling pathway activation in DN, a process not previously reported to be involved in this disease.

## Introduction

DN is a complex metabolic disease that evolves into a progressive fibrosing kidney disease. It represents the leading cause of end-stage renal failure in the industrialized world. A series of pathogenic mechanisms have been shown to contribute to this chronic progressive disease including: hyperglycemia with advanced glycosylation end products, hemodynamic and vascular alterations with albuminuria, and the intrarenal production of growth factors and matrix components [1]. In recent years it also became evident that inflammatory mechanisms contribute significantly to the development and progression of DN. These include the infiltration of renal compartments by lymphocytes and monocytes/macrophages as well as local production of cytokines and chemokines in the kidney (recently summarized in [2], [3]). Specific inflammatory and angiogenic molecular pathways have been recently linked to progressive DN by the analysis of human renal gene expression profiles [4], [5].

While morphometric analysis of renal biopsy samples can demonstrate an inflammatory infiltrate in most advanced DN samples, it has been difficult to reliably characterize inflammatory features at the transcriptomic level suggesting insufficient resolution or sensitivity of oligonucleotide microarrays or of the data analysis.

This uncertainty in reliable detection of gene expression is thought to be associated with several issues connected to oligonulceotide microarrays such as Affymetrix DNA chips. First, the current annotation associated with a considerable number of probe sets may not be in line with current knowledge of the genome [6], [7]. Second, the current approach of averaging oligonucleotide signals in a gene-oriented way impairs the resolution of splice isoforms or alternative transcripts. Signals from cross-hybridizing probes are averaged into the probe set calculation leading to increased “noise” in the analysis. Researchers have attempted to address these problems by re-defining probe sets accordingly [6]. However, the pace of continued discovery of additional alternative mRNA transcripts and the constant refinement of gene annotation has demonstrated that such improvements were temporary, and can propagate the basic problems of probe set annotation. These issues are directly relevant for accurate analysis of array experiments independent of any particular statistical methods used.

We choose to apply two existing program packages in order to elucidate how well each of these methods correlates the same transcriptomic data with the observed biology. One, Robust Multi-array Analysis (RMA) is based on Affymetrix probe sets, while the other, ChipInspector (CI), is based on single probe analysis, bypassing probe set definitions entirely. In theory, the CI approach should provide a reduced level of experimental “background noise” with potentially enhanced sensitivity.

To test this hypothesis, samples taken from human renal tissue with or without DN were compared and contrasted using conventional and single probe analysis. A common data set of CEL files (Affymetrix HG-U133A) was analyzed using standard techniques (RMA) followed by significance analysis of microarrays (SAM) [8]. In parallel single probe analysis was performed using default settings (CI also using SAM) and yielded comparable numbers of regulated genes. However, gene ontology analysis by Database for Annotation, Visualization and Integrated Discovery (DAVID) [9] of both data sets showed a clearly improved representation of biological processes linked to the development and progression of DN for the single probe-based approach. The single probe methodology allowed the unique detection of Wnt-pathway activation in DN.

## Results

### Comparison of prior microarray studies with biological phenomena indicates the need for increased sensitivity in array analysis

**Figure 1:**
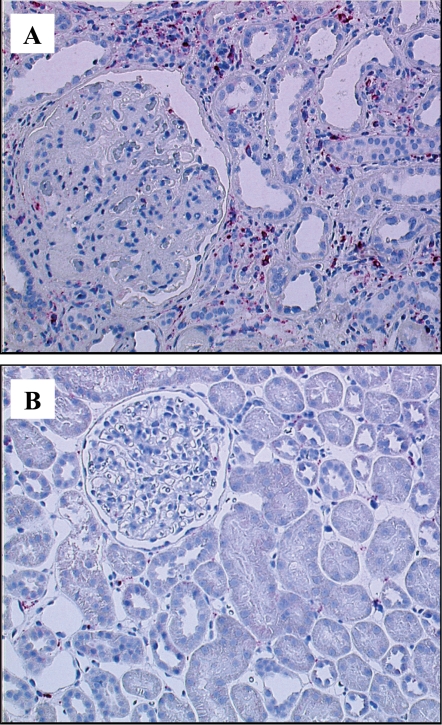
Tubulo-interstitial CD68^+^ cell infiltrate in DN. (a) In DN a prominent infiltration of CD68^+^ cells (monocytes/macrophages, stained in red) is observed in the tubulo-interstitium. A glomerulus showing nodular glomerulosclerosis (Kimmelstiel-Wilson), a classical histological sign for DN, has no prominent infiltrate. In control tissue (b) only few cells are CD68^+^. Together with the staining for CD3 and CD8 (shown in Table S1) this demonstrates the significant inflammatory reaction in DN.

Inflammatory processes may underlie important events in the pathogenesis of DN [10]. We previously demonstrated activation of the inflammatory transcriptional regulators nuclear factor-kappa B (NFκB) and interferon regulatory factor (IRF) linked to the progression of DN [4]. This observation prompted us to look more closely for evidence of inflammatory events in DN. Biopsy samples from patients presenting with advanced DN were examined by immunohistochemistry for specific inflammatory cell types. Staining for T cells (markers CD3, CD8), B cells (CD45RA) and monocytes/macrophages (CD68) showed a prominent infiltration in the renal tubulo-interstitium of patients with advanced DN (**Fig. 1** and **supplemental data**, **Table S1**). Although histological characterization clearly demonstrated inflammatory processes at work in samples of advanced DN, RMA based array and gene ontology (GO) analysis could identify only limited regulation of GO categories associated with inflammation suggesting a more sensitive approach was needed [4].

### Parallel array analysis by probe set- and single probe-based approaches

**Figure 2:**
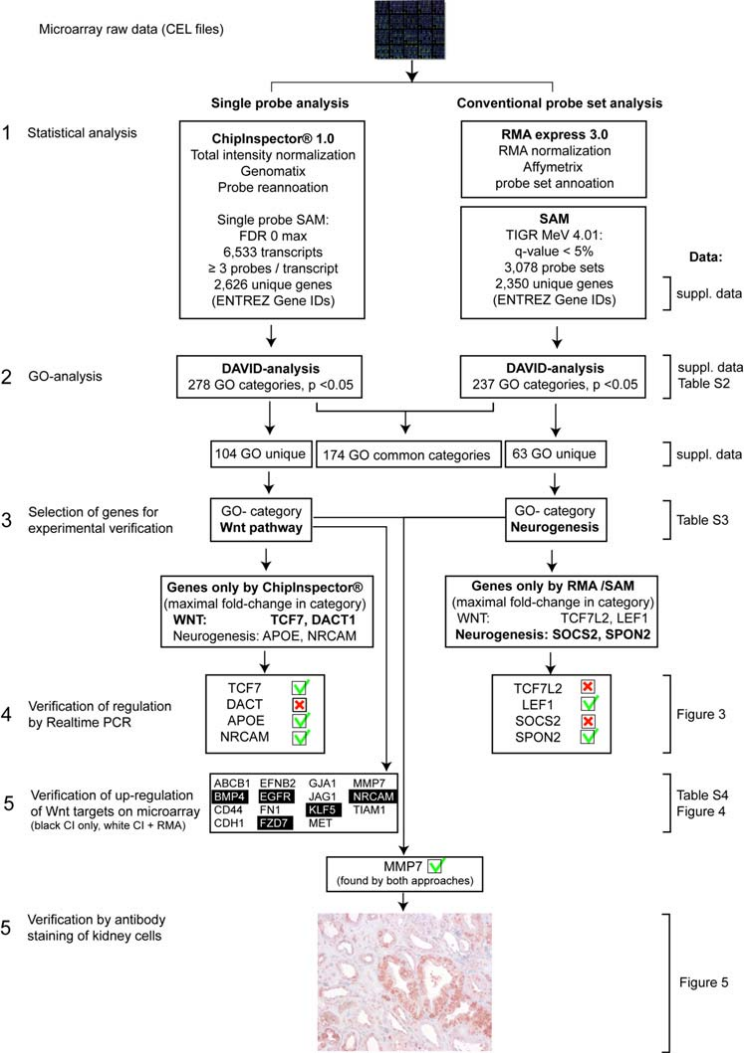
Schematic overview. The strategy of analysis and verification is depicted. Links to the data for each step are provided on the right. All genes where expression changes were predicted and verified by RT-PCR bear a green check mark; if not confirmed by RT-PCR, a red cross-mark is shown. In Step 5 only genes are shown that were both found significantly upregulated (white = both approaches, black CI only) and known Wnt pathway target genes.

Affymetrix DNA array data were analyzed in parallel using single probe-based analysis (CI) and conventional probe set-based algorithms (RMA). Expression profiles (CEL files) from microdissected tubulo-interstitial regions from patients with advanced DN (n = 6) and kidneys without functional alterations from living donors (LD, n = 3) were used in the subsequent analysis (see **Fig. 2** for schematic overview).

The microarray HG-U133A used in this study contains 195,294 single oligonucleotide probes with different sequences (control mismatch probes not included). These probes are used to generate 22,283 probe sets annotated by the manufacturer to 12,742 genes (Affymetrix, version 22).

The probe set-based analysis using RMA normalization identified 10,698 probe sets (48%) expressed above background. Significance analysis using SAM detected differential regulation of 2,350 unique genes, corresponding to 3,078 probe sets (q-value<5%, multiple comparison analysis of DN and controls as determined in previous studies, see methods).

After elimination of probes that could cross-hybridize to other transcripts, CI using the identical CEL files identified 39,933 significantly regulated individual probes using the default settings (see methods for details). With default settings of a minimum of 3 probes matching to a *de novo* annotated transcript, 6,533 transcripts were found to be significantly regulated, corresponding to 2,626 genes.

The initial analysis summarized in **Fig. 2** identified 1,466 genes found in common by both approaches. CI uniquely identified 1,160 genes and 884 genes were unique to the RMA analysis. While this shows a solid common core of regulated genes, gene lists per se are not suitable for evaluation of the biologically meaningful differences between the resulting lists. As our goal was to identify biological processes involved in DN, we mapped the genes from each approach onto GO. A relative ranking of the association of the various GO-categories with respect to the gene lists was carried out employing DAVID. The DAVID tool was developed for GO-ranking, and is independent of methodological differences between the microarray analyses tools used in this study. DAVID assigns a p-value to each biological process associated with the gene lists. As both methods (RMA, CI) yielded comparable numbers of genes under the conditions used, results from the DAVID analysis could be used to directly compare the methods.

### Single probe-based analysis identifies more specific functional categories than the probe set-based analysis

DAVID analysis of all regulated genes and transcripts found by the two analysis techniques yielded 174 common GO-categories (supplemental data, **Table S2**; cut-off p-value 0.05) again demonstrating the significant common core of both data sets. The CI-based associations showed overall lower p-values in these common 174 GO-categories (lower p-value in 137 of 174 categories). For the sake of comparison only significant (p<0.05) categories were used in subsequent analyses. DAVID found an additional 104 significant GO-categories uniquely associated with the CI-derived gene list, while the RMA-derived list matched uniquely to 63 additional significant GO-categories (**supplemental data**, **Table S3**).

As the study was initiated by a search for inflammatory gene signatures involved in DN, we compared functional categories covering inflammatory mechanisms. Both methods identified seven common inflammation-associated GO-categories (*GO:0019883*, *GO:0019885*, *GO:0006956*, *GO:0006958*, *GO:0002455*, *GO:0006959*, *GO:0045087*; **Table S2**). Importantly, CI analysis listed 13 additional GO-categories not found by the RMA analysis (*GO:0019730*, *0006952*, *0045321*, *0006955*, *0006954*, *0046649*, *0050778*, *0051251*, *0050870*, *0051249*, *0050863*, *0009605*, *and 0042110*; see **Table S3**). No inflammation-associated GO-classification was found uniquely by the probe set-based approach.

In a recent report we described the regulation of angiogenesis-associated genes in DN, including the down-regulation of the vascular endothelial growth factor A (VEGF-A) [5]. Comparing angiogenesis-associated GO-categories showed that the gene list generated by CI included significant counts for *“angiogenesis” (GO:0001525)*, *“blood vessel development” (GO:0001568)*, *“blood vessel morphogenesis” (GO:0048514)*, and *“vasculature development” (GO:0001944)*. In contrast, no significant angiogenesis-associated categories were identified by RMA analysis (**Table S3**).

### Functional categories uniquely found by each approach and confirmatory studies

Each of the approaches uniquely identified GO-categories, although individual genes belonging to these categories were identified by each method. To analyze the results in more detail, we then selected genes from potentially relevant functional categories not been previously reported in DN. In this regard, neurogenesis identified by RMA-based analysis, and the Wnt signaling pathway found by the CI approach were chosen for further analysis.

**Figure 3:**
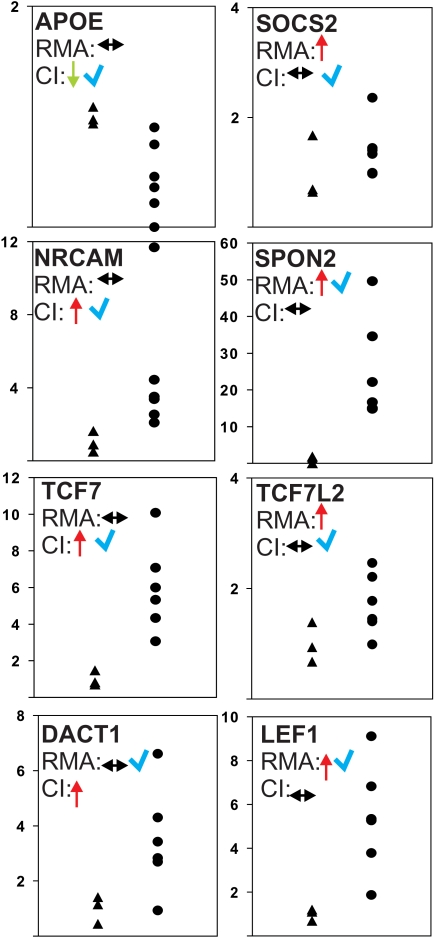
Validation of the array expression pattern by real-time RT-PCR. The expression of selected transcripts predicted by CI or RMA, respectively, was tested by real-time RT-PCR. The same cDNA hybridized on the array was used. Expression is shown for controls (living donors (LD), n = 3, triangles) and DN (n = 6, full circles). In the upper left corner gene symbols and predictions by each method are indicated (red arrow: induced, green arrow: reduced, black arrow: not regulated, compared to controls). Blue check indicates confirmation of the respective prediction. The results in detail: Genes associated with neurogenesis: APOE (predicted by CI) LD 1.00±0.08, DN 0.45±0.33 (p<0.05); SOCS2 (predicted by RMA) LD 1.00±0.58, DN 1.42±0.50 (n.s.); NRCAM (CI) LD 1.00±0.57, DN 4.61±3.56 (p<0.05); SPON2 (RMA): LD 1.00±0.92, DN 25.47±13.98 (p<0.05). Genes associated with Wnt signaling: TCF7 (predicted by CI): LD 1.00±0.41, DN 5.98±2.49 (p<0.05); TCFL7 (predicted by RMA): LD 1.00±0.36, DN 1.72±0.55 (n.s.); DACT1 (CI): 1.00±0.50, DN 3.47±1.894 (n.s.); LEF1 (RMA): LD 1.00±0.27, DN 5.37±2.49 (p<0.05).

“Neurogenesis” was represented by six GO-categories in the RMA-based gene lists (*GO:0048813*, *GO:0048812*, *GO:0022008*, *GO:0048666*, *GO:0030182*, and *GO:0048667*) but absent as GO category in the single probe analysis. The unique lists contained 1, 6, 9, 7, 8, and 6 genes for RMA in above GO categories and 0, 5, 5, 5, 5, and 5 genes for CI, respectively (see **Table S3** and **supplemental data**, **Table S4**). RT-PCR analysis of the original cDNA used for hybridization was used to verify expression of the two genes showing the highest fold-change in the lists of neurogenesis-associated genes uniquely found by probe set- and single probe-based approaches. SOCS2 and SPON2 were selected from the RMA-list of genes, for the CI-generated list the genes APOE and NRCAM were tested (**Table S3** and **supplemental data**, **Table S5a**). Expression was confirmed for transcripts of APOE (predicted by CI), NRCAM (CI), and SPON2 (RMA), while SOCS2 (RMA) could not be verified by RT-PCR (**Fig. 3** and **Table S5a**).

The Wnt signaling pathway (*GO:0016055*) was identified only in DAVID analysis of CI-derived gene list, but significantly regulated genes from the Wnt pathway were identified by both approaches. CI matched 14 genes uniquely while RMA identified 5 genes uniquely. Again the genes showing the highest fold change in each approach-specific gene list were further analyzed by RT-PCR (TCF7L2 and LEF1 for RMA and TCF7 and DACT1 for CI) (**Table S3** and **Table S5a**). The mRNA expression was confirmed for the TCF7 (CI) and LEF1 (RMA) transcripts. The predictions of DACT1 (CI) and TCF7L2 (RMA) could not be verified by RT-PCR (**Fig. 3**).

The number of probes with ambiguous matches is listed in supplemental **Table S5b** for each of the selected probe set. Ambiguous matches were found in TCF7 and TCF7L2, the later, a gene recently linked to the development of diabetes (but not DN) [11], [12]. Importantly, the probe set 216511_s_at for TCF7L2 was found to map downstream of all three transcripts annotated for TCF7L2. Two additional probe sets for TCF7L2 mapped to exons and were not listed as regulated in DN by either approach (see **Table S5a and b**).

### Wnt signaling in diabetic nephropathy

**Figure 4:**
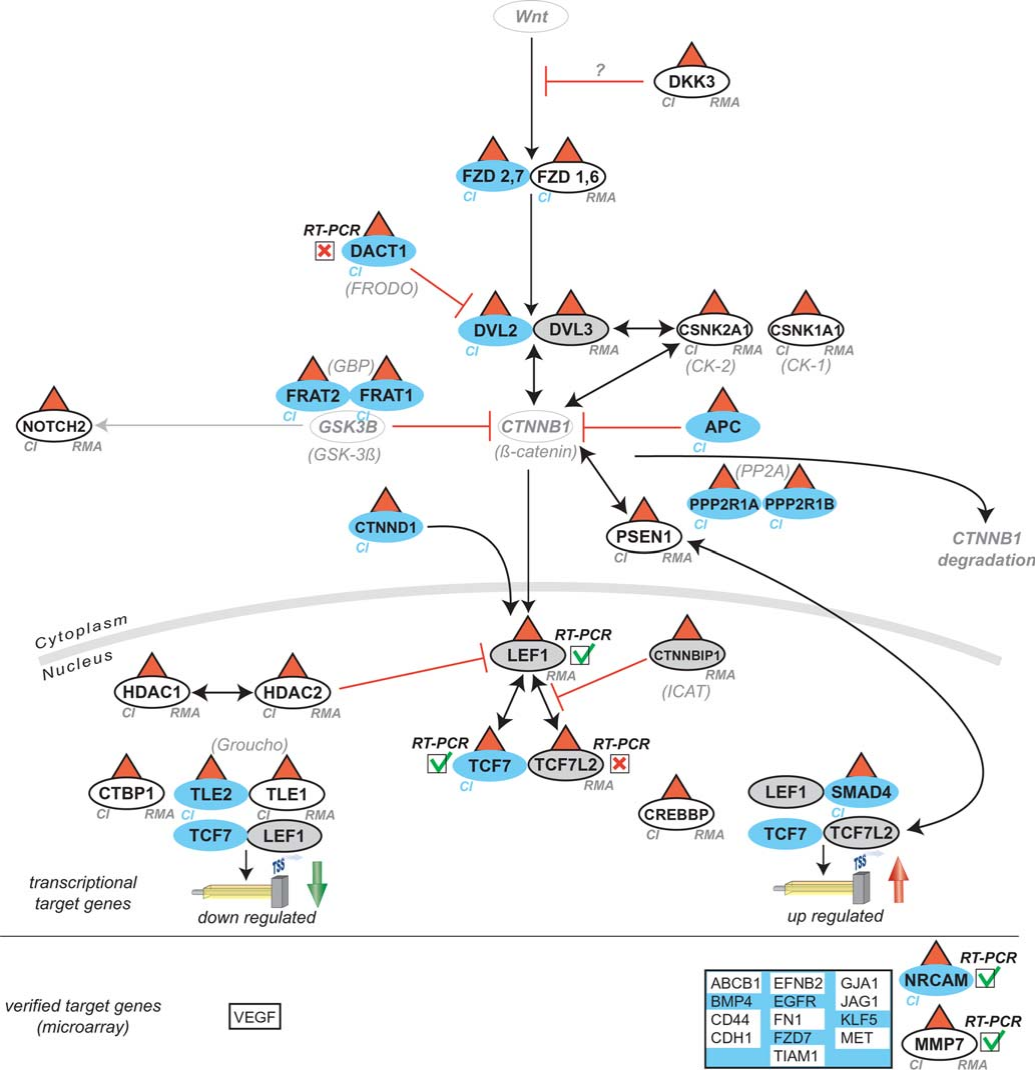
Mapping of respective genes found by CI or RMA onto the canonical Wnt-pathway. A scheme of the Wnt-pathway is depicted. Black arrows indicate activation and red lines inhibition. Genes found only by CI are annotated with CI with a blue background (n = 13, 2 genes [NRCAM, TCF7] confirmed by real-time RT-PCR, 1 gene failed [DACT1]), while genes only found by the RMA approach have a grey background with a black frame and are marked with RMA (n = 4, only 1 gene [LEF1] confirmed by real-time RT-PCR, 1 gene [TCF7L2] failed). Genes found by both approaches are annotated with CI and RMA and on a white background (n = 12, 1 gene confirmed by RT-PCR [MMP7]). A red triangle indicates up-regulation of the respective gene. Genes which were not found by any array analysis but are important for the comprehension of the basic pathway have a grey font. Comparison of known Wnt-target genes resulted in 15 genes specifically regulated in the microarray analysis (below the horizontal line). The respective genes found only by CI (n = 5) have a blue background (1 gene [NRCAM] confirmed by real-time RT-PCR), while the genes (n = 10) found by both approaches have a white background. VEGF was the only gene found to be down-regulated. There were no Wnt target genes only found by RMA. Gene symbols are according to HUGO, synonyms are shown in brackets.

**Figure 5:**
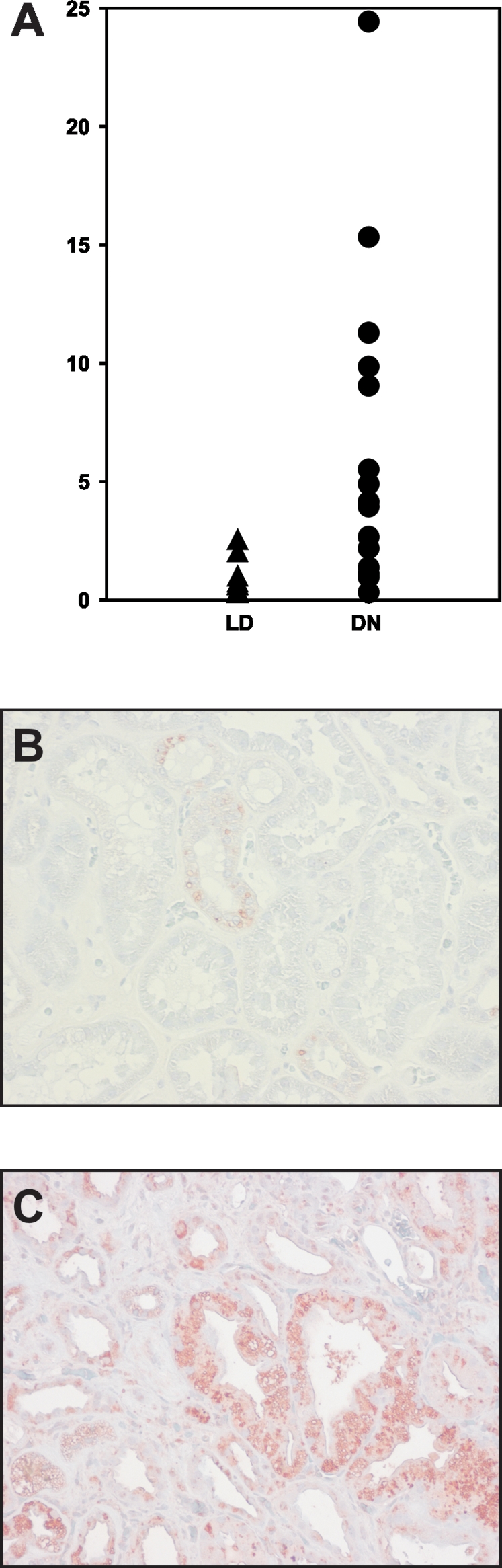
MMP7 mRNA expression is increased in DN. (a) MMP7 mRNA was quantified by RT-PCR on an independent cohort and showed an induction in DN (DN (n = 16): 6.45±6.62; LD (n = 9): 1.00±0.79 (p<0.05)). MMP7 protein (marked in red) is also induced in DN as shown by immunohistochemistry. (b) Control kidneys show limited expression of MMP7 protein in tubular epithelial cells. (c) In DN, MMP7 is markedly upregulated in tubular epithelial cells and in the interstitial compartment.

The potential relevance of Wnt signaling in advanced DN was investigated in more detail. Mapping the respective genes found by each approach onto the canonical Wnt pathway was performed (KEGG [13] and Biocarta databases (BioCarta Pathways; http://www.biocarta.com/genes/index.asp)). As shown in **Fig. 4**, and in line with previous findings, the CI-analysis identified a much larger fraction of the pathway as regulated than did the RMA analysis (23 versus 15 out of 27 genes, see **Table S3** and **Table S4**). The potential downstream effects of this pathway on known Wnt target genes were then examined. Of the known Wnt target genes regulated on the microarray 15 of 15 were identified by CI while RMA identified 10 (**Fig. 4** and **Table S4**). Matrix metalloproteinase 7 (MMP7) [14] showed the highest fold-change in Wnt-associated genes and was confirmed by RT-PCR on the cDNA used for the array analysis (DN 40.09±23.88, LD: 1.0±1.73 (p<0.05)) as well as on an independent cohort of patients with DN (DN: 6.45±6.62; LD: 1.00±0.79 (p<0.05)) (**Fig. 5a**). The induction of MMP7 protein was verified by immunohistochemistry: MMP7 protein expression was strongly increased in the tubulo-interstitial compartment of patients with DN (**Fig. 2** and **Fig. 5b,c**)

## Discussion

DN develops gradually in response to systemic metabolic changes and the cellular responses to these effects. The molecular mechanisms that underlie progression of the disease to end stage renal failure are not well defined thus limiting access to potential therapeutic targets.

In contrast to the immune reaction in response to acute infection or inflammation, the immune processes in chronic diseases such as DN can be “smoldering” processes that are hard to detect. The pathophysiologic effects elicited by such an immune cell infiltrate lead to the accumulation of subtle damage over a long period of time [10]. Understanding the small changes in the biological networks that regulate these processes is central to characterizing the underlying pathogenetic events. Elucidation of the regulatory networks driving the tissue damage could thus improve the diagnosis in what can now be seen a heterogeneous disease and may help to identify potential targets for therapeutic intervention.

Microarrays are widely applied for the characterization of transcriptomic changes in diseased tissues. However, in previous array analyses the approach has failed to detect some of the subtle changes that occur in patient samples associated with the development of chronic disease [4], [5]. Lowering thresholds for the detection of significantly regulated genes using standard analysis approaches results in an increase in “background noise” in the experiment and increasingly unreliable results [15].

**Figure 6:**
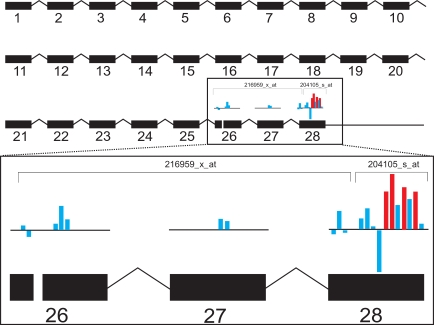
Graphical overview of the gene locus of NRCAM. A schematic view of the NRCAM gene structure (NM_005010) is shown consisting of 28 exons (black rectangles). Probes of the respective probe sets are indicated as blue or red bars. Red bars indicate probes with significantly higher signal intensity in DN samples compared to controls; blue bars represent probes indicating no induction or repression. The height of each bar indicates the fold-change for each single probe.

Previous reports have outlined the potential advantages of using a single probe-based approach for gene annotation and expression analysis [16]. Therefore, we directly compared a single probe-based method (CI) with a common probe set-based method (RMA). The overall picture that emerged from the CI analysis was more inline with the pathologic observations. After confirmatory RT-PCR studies the analysis of the probe signals suggested that differences in single probe versus probe set calculation were a major reason for the differences observed between the two analysis methods (RMA, CI). **Fig. 6** demonstrates the effect that basing calculations on single probes versus probe sets can have in the analysis. The difference between the signals of the individual probes mapped to NRCAM shows why this gene escaped detection based on an average value of all these probes by RMA. DACT1 predicted to be regulated by CI narrowly missed statistical significance at the confirmatory studies (p = 0.07) probably in part due to different statistical methods applied to array and RT-PCR results. While the results appeared to favor the single-probe approach a few genes were missed by CI due to insufficient coverage by unique mapped probes (LEF1) or because the probe annotations were not yet included in the database at the time of analysis (SPON2, see **Table S5b**).

The comparative analysis detailed here was based on probe annotation and aspects of the relevant underlying biology. The different statistics employed inside the methods will additionally influence the result. However, as the goal of this study was to improve the links between transcriptomic analyses and biological/clinical observations, it is irrelevant to what extent probe annotation and/or algorithmic differences are contributing to the performance of the two program packages. Overall association with biological processes was found to be improved for the single probe-based method as judged by the number of GO-categories and genes associated with relevant processes (inflammation and angiogenesis). Moreover, signaling pathways were easier to identify and superposition of the significantly regulated genes yielded overall a more complete picture provided by the single probe-based analysis.

In the course of the study the Wnt pathway was identified to be associated with DN. This important biological pathway has not been studied in detail in DN. The Wnt signaling pathway is known to play a role in renal development and cystogenesis [17]. The most highly regulated Wnt target gene identified in the analysis was MMP7. Diverse roles for matrix metalloproteinases have been postulated in various renal pathophysiologies [18]. A reduced expression of MMP7 has been recently described in rodent models of DN [19] but no human data on MMP7 expression in DN has been reported. Contrary to what has been reported in the rodent models, MMP7 was shown to be induced mainly in tubular epithelial cells in human DN. Discrepancies between the findings in rodent models for DN and human disease have been previously reported (e.g. [5], [20]). While the up-regulation of a matrix degradating protease in a setting of chronic fibrosis seems counterintuitive, the MMP family plays diverse roles in regulating tissue remodeling [21].

The RMA based approach uniquely identified neurogenesis processes that may also be relevant in DN. However, in contrast to the Wnt signaling pathway, neurogenesis was not represented by a single GO-category but was rather represented by six different processes. All but one of them also showed a robust, although not significant representation in the list of unique genes from CI (**Table S3** and **Table S4**; respective SPON2 see **Table S5b**). The potential finding of neurogenesis-associated processes in DN will need further verification and additional experiments in future.

A significant difference (p<0.05) in confirmatory RT-PCR experiments using the same cDNA as for the microarray hybridization served as a stringent paramenter for microarray analysis evaluation. However, as group sizes were limited, gene regulation in DN of some genes defined as “false positives” can not be excluded.

In summary, the single probe based analysis of oligonucleotide based arrays demonstrated clear advantages over the common probe set-based approach used in this study, most notably resulting in improved sensitivity and specificity of the biological findings (DAVID analysis). This was highlighted by the unique detection of a number of categories directly linked to clinical observations (inflammation and angiogenesis). In addition, the highlighted involvement of the Wnt pathway was in line with a larger body of data from the same microarray (Wnt target genes, **Fig. 2** and **4**).

## Materials and Methods

### Kidney biopsies, micordissection, RNA isolation and target preparation

Human renal biopsies from controls and patients with DN were collected from the European Renal cDNA Bank - Kröner-Fresenius Biopsy Bank (ERCB-KFB), a multi-center study on renal gene expression in human nephropathies. Diagnostic renal biopsies were obtained from patients after informed consent and with approval of the local ethics committees. Microdissected samples taken from the tubulo-interstitial compartment were processed as described [22]. For oligonucleotide array based gene expression profiling of DN a total of 9 kidney biopsies from individual patients were included: Biopsies from patients with advanced DN (n = 6) were analyzed and compared with pre-transplantation kidney biopsies from living donors as control renal tissue (LD, n = 3). For confirmation of MMP7 induction, predicted by both array analysis approaches, an additional independent cohort from the ERCB-KFB was analyzed (DN, n = 16; controls, n = 9). The biopsies were stratified by reference pathologists according to their histological diagnosis.

Following renal biopsy, the tissue was transferred to RNase inhibitor and microdissected into glomerular and tubular fragments. Total RNA was isolated from microdissected tubulointerstitial tissue (for details see [22]).

300–800 ng of total RNA was reverse-transcribed (RT) and linearly amplified according to a protocol previously reported [4]. The fragmentation, hybridization, staining and imaging were performed according the Affymetrix Expression Analysis Technical Manual.

For microarray analysis Robust Multichip Analysis (RMA) was performed. Subsequently we analyzed the expression arrays with Significance Analysis of Microarrays (SAM) [8]. For more details and for gene expression data of respective probe sets see http://diabetes.diabetesjournals.org/cgi/content/full/55/11/2993.

### Microarray Data Analysis

To compare the respective analysis approaches both methods used the same original CEL files resulting from the Affymetrix Chip reader. Both program packages were used with either default or previously tested parameters.

#### I. Probeset-based analysis: Robust Multichip Average (RMA)

RMA (RMAexpress version 0.3) consists of three steps: background adjustment, quantile normalization, and summarization [23]. RMA utilizes the Affymetrix provided probe set annotation to identify genes directly from the CEL files. The following settings were taken from previous studies [4], [5]: a background filter cut-off was defined to lower the count of false positive calls using the highest signal value obtained from non-human Affymetrix control oligonucleotides multiplied by a factor of 1.2, corresponding in the current data set to a log based 2 value of 5.8. Following normalized RMA, SAM analysis software (TIGR MeV Version 4.01, http://www-stat.stanford.edu/tibs/SAM/) was applied using a q-value<5% to identify genes that were differently regulated between the analyzed groups.

#### II. Single probe-based analysis: ChipInspector (CI)

ChipInspector (version 1.3; Genomatix Software GmbH, Munich) is a novel single probe-based analysis tool for microarray data that consists of four steps: single probe-transcript annotation, total intensity normalization, SAM analysis (adapted to single probe handling) and transcript identification based on significantly changed probes. All probes on the array are individually matched against the appropriate genome and all known transcripts thereof available at the time of analysis. Only probes that match uniquely to the genome and to at least one transcript (or overlapping transcripts) are retained for further analysis. The input data for the SAM analysis in this case were single probe values and the resulting probes showing significantly changed signals are then used to identify the corresponding transcripts (which may be more than one per gene). Fold changes are not used and the expression ratios shown in the result table are calculated from the average expression levels of all significant probes of each individual transcript. Gene identifiers are then attached to each transcript.

SAM creates artificial background data by randomly permuting the array results. Each probe has a score on the basis of its fold change relative to the standard deviation of repeated measurements for this probe. Probes with scores higher than a certain threshold are deemed significant. This threshold is the Delta value. The permutations of the data set are then used to estimate the percentage of probes identified by chance at the identical Delta. Thus, a relation of significant probes to falsely discovered probes can be given for each Delta threshold. This relation is the False Discovery Rate (FDR), a stringency indicator. Analysis was carried out using all default settings as recommended by the software provider, except for the expected FDR, which was set to maximal detection of regulated transcripts with lowest amount of falsely called features (FDR 0%).

### Ranking of RMA and CI results by GeneOntologyChart

The GOChart in DAVID (version 2007; [9]) was used to establish the distribution of differentially regulated genes attributed to functional biologic categories for both resulting gene lists. The controlled hierarchical vocabulary of the Gene Ontology Consortium provides a structured language that can be applied to the functions of genes and proteins in all organisms [24]. The biological theme determination of gene lists in DAVID are based on the Expression Analysis Systematic Explorer (EASE), a variant of one-tailed Fisher exact probability [25]. At the time of analysis multiple comparison correction was not implemented in DAVID because it has been considered to be too conservative and might hurt biology (communication of DAVID, 24 March 2007). For DAVID analyses a p-value of 0.05 was used as standard cut-off level.

### RT-PCR analysis used for validation

Reverse transcription and real-time RT-PCR was performed as reported earlier [22]. Pre-developed TaqMan reagents were used for human APOE, DACT1, LEF1, NRCAM, SOCS2, SPON2, and 18S rRNA (Applied Biosystems). For TCF7 and TCF7L2 the following oligonucleotide primers (300 nmol/L) and probe (100 nmol/L) were used: human TCF7, sense primer 5′-TCAGGGAAGCAGGAGCTG-3′, antisense primer 5′-TTCTTGATGGTTGGCTTCTTG-3′; fluorescence labeled probe (FAM) 5′-ACCGCAACCTGAAGACACAAGCAGA-3′, human TCF7L2 sense primer 5′-GGATTCAGACACCCCTACCC-3′, antisense primer 5′-CGTGTGTAGCGTATGATGTGG-3′; fluorescence labeled probe (FAM) 5′-CAATGCTTCCATGTCCAGGTTCCCT-3′. The expression of candidate genes was normalized to the reference gene 18SrRNA showing robust expression in human tubulo-interstitial tissue samples [26]. The mRNA expression was analyzed by standard curve quantification.

### Immunohistochemistry

Immunohistochemistry for T cells, B cells and monocyte/macrophages was performed essentially as described [27]. The MMP7 monoclonal antibody (Thermo Scientific, Fremont, CA, #MS-813-R7) was used according to the manufactures directions at a dilution of 1∶20.

### Statistics used for RT-PCR result evaluation

Data are given as mean±SD. Statistical analyses were performed using SPSS 16.01 (SPSS Inc., Chicago, IL). Significance in immunohistochemical staining was evaluated using Kruskal-Wallis and Mann-Whitney U tests. The non-parametric two-sample test of Kolmogorov-Smirnov as well as the Moses-test were applied to the real-time RT-PCR analyses of eight selected candidate genes. A p-value<0.05 indicates a statistically significant difference.

## Supporting Information

Table S1 Quantification of immunohistochemical staining for cell infiltrate. Renal biopsy tissue was stained for the T-cell markers CD3, CD8, B-cells (CD45RA) and monocytes/macrophages (CD68). In DN (n = 7) CD3+ cells, CD8+ cells, overall T-cells and B-cells and CD68+ cells were more frequently observed than in controls (n = 4) (*: p<0.05).
(0.04 MB DOC)

Table S2 Prominent biological aspects found by DAVID analysis in both ChipInspector and RMA output lists. Shown are the 174 biological aspects found by both approaches. Gene ontology categories found only by one of both approaches are listed in Table S3.
(0.44 MB DOC)

Table S3 DAVID analysis of all regulated genes and transcripts found by the two independent array analysis techniques. Shown are the prominent biological aspects found by CI only (“Class 1”) or RMA only (“Class 2”). In total 174 common GO-categories were found (see, Table S2), additional 104 GO-categories were uniquely associated with the CI-derived gene list, while 63 additional GO-categories were found by RMA only.
(0.35 MB DOC)

Table S4 Gene list of Wnt receptor signaling pathway, neurogenesis, and Wnt target genes. Genes of the selected GO categories are shown with their respective regulation indicated by either analysis method. Bold are the gene symbols of genes with the highest fold changes, which were selected for confirmatory studies. In addition, Wnt target genes are listed. Only fold changes indicated as significant by the respective method are shown.
(0.34 MB DOC)

Table S5 Probe set information and analysis results for the genes selected for confirmatory studies. A) Results of the analyses performed with CI and RMA are shown. Fold change and significance are given for all transcripts (for CI) or probe sets (for RMA) indicated by one of both methods to be significantly regulated. * indicates significance, n.s. = not significant, BC = expression below cut-off (see method section). SPON2 was missed by the initial version of CI (see above). B) Shown are the number of probes in a probe set, number of probes with a perfect and with a unique match, and the number of probes mapping to an exon. The information has been extracted from Eldorado (Genomatix, Germany). Accession numbers for known transcripts and probe set identification numbers are listed. SPON2 was not annotated in Eldorado at the time of analysis. The latest version gives results indicated as “corrected”.
(0.15 MB DOC)

## References

[1] Schena FP, Gesualdo L (2005). Pathogenetic mechanisms of diabetic nephropathy.. J Am Soc Nephrol.

[2] Ruster C, Wolf G (2008). The role of chemokines and chemokine receptors in diabetic nephropathy.. Front Biosci.

[3] Navarro-Gonzalez JF, Mora-Fernandez C (2008). The role of inflammatory cytokines in diabetic nephropathy.. J Am Soc Nephrol.

[4] Schmid H, Boucherot A, Yasuda Y, Henger A, Brunner B (2006). Modular activation of nuclear factor-kappaB transcriptional programs in human diabetic nephropathy.. Diabetes.

[5] Lindenmeyer MT, Kretzler M, Boucherot A, Berra S, Yasuda Y (2007). Interstitial vascular rarefaction and reduced VEGF-A expression in human diabetic nephropathy.. J Am Soc Nephrol.

[6] Dai M, Wang P, Boyd AD, Kostov G, Athey B (2005). Evolving gene/transcript definitions significantly alter the interpretation of GeneChip data.. Nucleic Acids Res.

[7] Elo LL, Lahti L, Skottman H, Kylaniemi M, Lahesmaa R (2005). Integrating probe-level expression changes across generations of Affymetrix arrays.. Nucleic Acids Res.

[8] Tusher VG, Tibshirani R, Chu G (2001). Significance analysis of microarrays applied to the ionizing radiation response.. Proc Natl Acad Sci U S A.

[9] Dennis G, Sherman BT, Hosack DA, Yang J, Gao W (2003). DAVID: Database for Annotation, Visualization, and Integrated Discovery.. Genome Biol.

[10] Galkina E, Ley K (2006). Leukocyte recruitment and vascular injury in diabetic nephropathy.. J Am Soc Nephrol.

[11] Florez JC, Jablonski KA, Bayley N, Pollin TI, de Bakker PI (2006). TCF7L2 polymorphisms and progression to diabetes in the Diabetes Prevention Program.. N Engl J Med.

[12] Grant SF, Thorleifsson G, Reynisdottir I, Benediktsson R, Manolescu A (2006). Variant of transcription factor 7-like 2 (TCF7L2) gene confers risk of type 2 diabetes.. Nat Genet.

[13] Kanehisa M, Goto S (2000). KEGG: kyoto encyclopedia of genes and genomes.. Nucleic Acids Res.

[14] Brabletz T, Jung A, Dag S, Hlubek F, Kirchner T (1999). beta-catenin regulates the expression of the matrix metalloproteinase-7 in human colorectal cancer.. Am J Pathol.

[15] Pawitan Y, Michiels S, Koscielny S, Gusnanto A, Ploner A (2005). False discovery rate, sensitivity and sample size for microarray studies.. Bioinformatics.

[16] Lu J, Lee JC, Salit ML, Cam MC (2007). Transcript-based redefinition of grouped oligonucleotide probe sets using AceView: high-resolution annotation for microarrays.. BMC Bioinformatics.

[17] Schedl A (2007). Renal abnormalities and their developmental origin.. Nat Rev Genet.

[18] Catania JM, Chen G, Parrish AR (2007). Role of matrix metalloproteinases in renal pathophysiologies.. Am J Physiol Renal Physiol.

[19] McLennan SV, Kelly DJ, Schache M, Waltham M, Dy V (2007). Advanced glycation end products decrease mesangial cell MMP-7: a role in matrix accumulation in diabetic nephropathy?. Kidney Int.

[20] Breyer MD, Bottinger E, Brosius FC, Coffman TM, Fogo A (2005). Diabetic nephropathy: of mice and men.. Adv Chronic Kidney Dis.

[21] Logan CY, Nusse R (2004). The Wnt signaling pathway in development and disease.. Annu Rev Cell Dev Biol.

[22] Cohen CD, Frach K, Schlondorff D, Kretzler M (2002). Quantitative gene expression analysis in renal biopsies: a novel protocol for a high-throughput multicenter application.. Kidney Int.

[23] Bolstad BM, Irizarry RA, Astrand M, Speed TP (2003). A comparison of normalization methods for high density oligonucleotide array data based on variance and bias.. Bioinformatics.

[24] Ashburner M, Ball CA, Blake JA, Botstein D, Butler H (2000). Gene ontology: tool for the unification of biology. The Gene Ontology Consortium.. Nat Genet.

[25] Hosack DA, Dennis G, Sherman BT, Lane HC, Lempicki RA (2003). Identifying biological themes within lists of genes with EASE.. Genome Biol.

[26] Schmid H, Cohen CD, Henger A, Irrgang S, Schlondorff D (2003). Validation of endogenous controls for gene expression analysis in microdissected human renal biopsies.. Kidney Int.

[27] Henger A, Kretzler M, Doran P, Bonrouhi M, Schmid H (2004). Gene expression fingerprints in human tubulointerstitial inflammation and fibrosis as prognostic markers of disease progression.. Kidney Int.

