# Supplementary material for: Improved Elucidation of Biological Processes Linked to Diabetic Nephropathy by Single Probe-Based Microarray Data Analysis
Source: PLoS One. 2008 Aug 13;3(8):e2937. doi: 10.1371/journal.pone.0002937 (PMC2493035; doi:10.1371/journal.pone.0002937)
Supplement: Table S1 — Quantification of immunohistochemical staining for cell infiltrate. Renal biopsy tissue was stained for the T-cell markers CD3, CD8, B-cells (CD45RA) and monocytes/macrophages (CD68). In DN (n = 7) CD3+ cells, CD8+ cells, overall T-cells and B-cells and CD68+ cells were more frequently observed than in controls (n = 4) (*: p<0.05). (0.04 MB DOC) [file pone.0002937.s001.doc]

##### Table S1: Quantification of immunohistochemical staining for cell infiltrate

Renal biopsy tissue was stained for the T-cell markers CD3, CD8, B-cells (CD45RA) and monocytes/macrophages (CD68). In DN (n=7) CD3^+^ cells, CD8^+^ cells, overall T-cells and B-cells and CD68^+^ cells were more frequently observed than in controls (n=4) (*: p<0.05).

|  | **CD3** | **CD8** | **T cells** | **B cells** | **CD68** |
| --- | --- | --- | --- | --- | --- |
| Control 1 | 0.25 | 1.66 | - | 0 | 0.33 |
| Control 2 | 0.2 | 0.77 | 6 | 1 | 0 |
| Control 3 | 0.78 | 0.25 | 10.69 | 1.81 | 3.92 |
| Control 4 | 0.14 | 0.2 | 6.16 | 0.4 | 0.16 |
| **mean ± sd** | **0.3 ± 0.3** | **0.7 ± 0.7** | **7.6 ± 2.7** | **0.8 ± 0.8** | **1.1 ± 1.9** |
|  |  |  |  |  |  |
| DN1 | 26.77 | 15.22 | 54.72 | 19.54 | 26.21 |
| DN2 | 26.6 | 22.95 | 80.81 | 18 | 31.16 |
| DN3 | 0.68 | 5.72 | 13.05 | 8.26 | 18.04 |
| DN4 | 23.37 | 16.12 | 63.65 | 32.94 | 22.56 |
| DN5 | 28.68 | 19.41 | 54.66 | 12.58 | 17.45 |
| DN6 | 5.14 | 4.3 | 13.65 | 1.75 | 9.14 |
| DN7 | 4.85 | 8.85 | 15.71 | 2.75 | 8 |
| **mean ± sd** | **16.6 ± 12.4 *** | **13.2 ± 7.1 *** | **42.3 ± 27.8 *** | **13.7 ± 10.9 *** | **18.9 ± 8.5 *** |
