# Supplementary material for: Improved Elucidation of Biological Processes Linked to Diabetic Nephropathy by Single Probe-Based Microarray Data Analysis
Source: PLoS One. 2008 Aug 13;3(8):e2937. doi: 10.1371/journal.pone.0002937 (PMC2493035; doi:10.1371/journal.pone.0002937)
Supplement: Table S2 — Prominent biological aspects found by DAVID analysis in both ChipInspector and RMA output lists. Shown are the 174 biological aspects found by both approaches. Gene ontology categories found only by one of both approaches are listed in Table S3. (0.44 MB DOC) [file pone.0002937.s002.doc]

**Table S2: Prominent biological aspects found by DAVID analysis in both ChipInspector and RMA output lists**

Shown are the 174 biological aspects found by both approaches. Gene ontology categories found only by one of both approaches are listed in Table S3.

| **Common GO categories** |  |  | **ChipInspector** | | | RMA/SAM | | |
| --- | --- | --- | --- | --- | --- | --- | --- | --- |
|  | GO Level | GO ID | **Genes involved in the GO category** | **involved genes/total genes**  **(%)** | **p-value (Ease-Score DAVID)** | **Genes involved in the GO category** | **involved genes/total genes**  **(%)** | **p-value (Ease-Score DAVID)** |
| **Carbohydrates** |  |  |  |  |  |  |  |  |
| carbohydrate biosynthesis | 4 | GO:0016051 | 22 | 0.84% | 3.32E-02 | 23 | 0.98 | 5.61E-03 |
| carboxylic acid metabolism | 4 | GO:0019752 | 103 | 3.93% | 3.81E-05 | 102 | 4.35 | 6.92E-07 |
| gluconeogenesis | >5 | GO:0006094 | 11 | 0.42% | 5.63E-03 | 9 | 0.38 | 2.79E-02 |
| hexose biosynthesis | >5 | GO:0019319 | 12 | 0.46% | 7.84E-03 | 11 | 0.47 | 1.10E-02 |
| monosaccharide biosynthesis | 5 | GO:0046364 | 12 | 0.46% | 7.84E-03 | 11 | 0.47 | 1.10E-02 |
|  |  |  |  |  |  |  |  |  |
| **Cell Cycle** |  |  |  |  |  |  |  |  |
| cell cycle | 2 | GO:0007049 | 158 | 6.02% | 3.25E-09 | 130 | 5.54 | 1.59E-05 |
| mitotic cell cycle | 3 | GO:0000278 | 41 | 1.56% | 2.28E-02 | 42 | 1.79 | 2.46E-03 |
| negative regulation of progression through cell cycle | 4, 5 | GO:0045786 | 41 | 1.56% | 1.08E-04 | 33 | 1.41 | 4.31E-03 |
| regulation of cell cycle | 3, 4 | GO:0051726 | 99 | 3.77% | 2.44E-05 | 87 | 3.71 | 2.13E-04 |
| regulation of progression through cell cycle | 3, 4 | GO:0051726 | 98 | 3.73% | 3.75E-05 | 86 | 3.66 | 3.18E-04 |
|  |  |  |  |  |  |  |  |  |
| **Cell death** |  |  |  |  |  |  |  |  |
| anti-apoptosis | >5 | GO:0006916 | 37 | 1.41% | 1.07E-05 | 37 | 1.58 | 9.35E-07 |
| apoptosis | 5 | GO:0006915 | 133 | 5.07% | 5.14E-10 | 113 | 4.81 | 3.74E-07 |
| cell death | 3, 5 | GO:0008219 | 139 | 5.30% | 1.30E-10 | 119 | 5.07 | 8.37E-08 |
| death | 2 | GO:0016265 | 140 | 5.34% | 1.08E-10 | 120 | 5.11 | 6.65E-08 |
| induction of apoptosis | >5 | GO:0006917 | 41 | 1.56% | 1.33E-05 | 27 | 1.15 | 4.02E-02 |
| induction of programmed cell death | 5 | GO:0012502 | 41 | 1.56% | 1.33E-05 | 27 | 1.15 | 4.02E-02 |
| negative regulation of apoptosis | 5 | GO:0043066 | 42 | 1.60% | 3.68E-06 | 42 | 1.79 | 2.37E-07 |
| negative regulation of programmed cell death | 4, 5 | GO:0043069 | 42 | 1.60% | 4.45E-06 | 42 | 1.79 | 2.91E-07 |
| positive regulation of apoptosis | 5 | GO:0043065 | 43 | 1.64% | 5.52E-05 | 30 | 1.28 | 3.69E-02 |
| positive regulation of programmed cell death | 4, 5 | GO:0043068 | 43 | 1.64% | 6.41E-05 | 30 | 1.28 | 3.95E-02 |
| programmed cell death | 4 | GO:0012501 | 133 | 5.07% | 6.56E-10 | 113 | 4.81 | 4.45E-07 |
| regulation of apoptosis | 4, 5 | GO:0042981 | 94 | 3.58% | 7.45E-10 | 79 | 3.37 | 5.37E-07 |
| regulation of programmed cell death | 3, 4, 5 | GO:0043067 | 94 | 3.58% | 1.02E-09 | 79 | 3.37 | 6.78E-07 |
|  |  |  |  |  |  |  |  |  |
| **Cytoskeleton** |  |  |  |  |  |  |  |  |
| actin cytoskeleton organization and biogenesis | >5 | GO:0030036 | 44 | 1.68% | 1.09E-05 | 35 | 1.49 | 1.30E-03 |
| actin filament polymerization | 5 | GO:0030041 | 11 | 0.42% | 2.16E-04 | 7 | 0.3 | 3.96E-02 |
| actin filament-based process | 5 | GO:0030029 | 45 | 1.71% | 2.80E-05 | 37 | 1.58 | 1.13E-03 |
| actin polymerization and/or depolymerization | 4 | GO:0008154 | 24 | 0.91% | 2.67E-08 | 15 | 0.64 | 2.14E-03 |
| cytoskeleton organization and biogenesis | 4 | GO:0007010 | 77 | 2.93% | 3.56E-03 | 64 | 2.73 | 4.05E-02 |
| regulation of actin filament length | 4 | GO:0030832 | 19 | 0.72% | 1.19E-06 | 12 | 0.51 | 6.89E-03 |
| regulation of actin polymerization and/or depolymerization | 4, 5 | GO:0008064 | 19 | 0.72% | 4.47E-07 | 12 | 0.51 | 4.45E-03 |
|  |  |  |  |  |  |  |  |  |
| **DNA/RNA** |  |  |  |  |  |  |  |  |
| chromatin modification | >5 | GO:0016568 | 29 | 1.11% | 7.35E-03 | 24 | 1.02 | 4.02E-02 |
| chromosome organization and biogenesis | 4 | GO:0051276 | 64 | 2.44% | 1.10E-03 | 54 | 2.3 | 1.13E-02 |
| chromosome organization and biogenesis (sensu Eukaryota) | 4 | GO:0007001 (GO:0051276) | 60 | 2.29% | 1.64E-03 | 50 | 2.13 | 1.87E-02 |
| establishment of RNA localization | 2, 3, 4 | GO:0051236 | 16 | 0.61% | 1.26E-03 | 14 | 0.6 | 4.14E-03 |
| mRNA export from nucleus | 5 | GO:0006406 | 12 | 0.46% | 7.84E-03 | 10 | 0.43 | 3.03E-02 |
| mRNA metabolism | 5 | GO:0016071 | 74 | 2.82% | 1.59E-11 | 63 | 2.68 | 9.73E-09 |
| mRNA processing | 3 | GO:0006397 | 65 | 2.48% | 3.80E-10 | 52 | 2.22 | 1.94E-06 |
| mRNA transport | 4, 5 | GO:0051028 | 13 | 0.50% | 4.15E-03 | 10 | 0.43 | 4.17E-02 |
| negative regulation of nucleobase, nucleoside, nucleotide and nucleic acid metabolism | 5 | GO:0045934 | 44 | 1.68% | 2.77E-04 | 38 | 1.62 | 1.88E-03 |
| negative regulation of transcription | 4, 5 | GO:0016481 | 41 | 1.56% | 6.22E-04 | 36 | 1.53 | 2.42E-03 |
| negative regulation of transcription from RNA polymerase II promoter | >5 | GO:0000122 | 18 | 0.69% | 1.82E-02 | 17 | 0.72 | 1.51E-02 |
| negative regulation of transcription, DNA-dependent | 5 | GO:0045892 | 28 | 1.07% | 3.85E-03 | 24 | 1.02 | 1.51E-02 |
| nuclear mRNA splicing, via spliceosome | 4 | GO:0000398 | 46 | 1.75% | 1.42E-09 | 37 | 1.58 | 2.12E-06 |
| nucleic acid transport | 4, 5 | GO:0050657 | 16 | 0.61% | 1.26E-03 | 14 | 0.6 | 4.14E-03 |
| nucleobase, nucleoside, nucleotide and nucleic acid transport | 3, 4 | GO:0015931 | 18 | 0.69% | 1.43E-03 | 14 | 0.6 | 2.20E-02 |
| positive regulation of transcription | 4, 5 | GO:0045941 | 26 | 0.99% | 2.60E-02 | 23 | 0.98 | 4.57E-02 |
| positive regulation of transcription, DNA-dependent | 5 | GO:0045893 | 22 | 0.84% | 1.38E-02 | 19 | 0.81 | 3.55E-02 |
| regulation of transcription from RNA polymerase II promoter | 5 | GO:0006357 | 71 | 2.71% | 3.94E-07 | 64 | 2.73 | 1.89E-06 |
| RNA catabolism | 5 | GO:0006401 | 12 | 0.46% | 1.48E-02 | 11 | 0.47 | 1.94E-02 |
| RNA export from nucleus | 5 | GO:0006405 | 15 | 0.57% | 2.37E-03 | 14 | 0.6 | 2.73E-03 |
| RNA localization | 3 | GO:0006403 | 16 | 0.61% | 1.60E-03 | 14 | 0.6 | 5.04E-03 |
| RNA metabolism | 4 | GO:0016070 | 121 | 4.61% | 3.90E-12 | 112 | 4.77 | 6.36E-12 |
| RNA processing | 2, 5 | GO:0006396 | 98 | 3.73% | 1.86E-10 | 92 | 3.92 | 9.97E-11 |
| RNA splicing | 3 | GO:0008380 | 54 | 2.06% | 2.02E-10 | 43 | 1.83 | 1.07E-06 |
| RNA splicing, via transesterification reactions | 4 | GO:0000375 | 46 | 1.75% | 1.42E-09 | 37 | 1.58 | 2.12E-06 |
| RNA splicing, via transesterification reactions with bulged adenosine as nucleophile | 5 | GO:0000377 | 46 | 1.75% | 1.42E-09 | 37 | 1.58 | 2.12E-06 |
| RNA transport | 3, 4, 5 | GO:0050658 | 16 | 0.61% | 1.26E-03 | 14 | 0.6 | 4.14E-03 |
| spliceosome assembly | 5 | GO:0000245 | 8 | 0.30% | 1.96E-02 | 8 | 0.34 | 1.13E-02 |
| telomere maintenance | 5 | GO:0000723 | 9 | 0.34% | 5.22E-03 | 8 | 0.34 | 1.13E-02 |
| transcription from RNA polymerase II promoter | 4 | GO:0006366 | 107 | 4.08% | 1.87E-06 | 96 | 4.09 | 9.06E-06 |
|  |  |  |  |  |  |  |  |  |
| **Immune response/Inflammation** |  |  |  |  |  |  |  |  |
| antigen processing and presentation of endogenous antigen | 3 | GO:0019883 | 18 | 0.69% | 9.62E-07 | 15 | 0.64 | 4.00E-05 |
| antigen processing and presentation of endogenous antigen via MHC class I | 5 | GO:0019885 | 19 | 0.72% | 7.39E-07 | 15 | 0.64 | 1.16E-04 |
| complement activation | 3, 4 | GO:0006956 | 13 | 0.50% | 3.25E-03 | 12 | 0.51 | 4.45E-03 |
| complement activation, classical pathway | 4, 5 | GO:0006958 | 10 | 0.38% | 1.10E-02 | 11 | 0.47 | 1.44E-03 |
| humoral immune response mediated by circulating immunoglobulin | 4 | GO:0002455 | 31 | 1.18% | 7.18E-04 | 25 | 1.07 | 1.20E-02 |
| humoral immune response | 3 | GO:0006959 | 44 | 1.68% | 2.89E-05 | 35 | 1.49 | 2.47E-03 |
| innate immune response | 3 | GO:0045087 | 20 | 0.76% | 2.19E-03 | 15 | 0.64 | 4.52E-02 |
|  |  |  |  |  |  |  |  |  |
| **Proteins/Enzymes** |  |  |  |  |  |  |  |  |
| amino acid and derivative metabolism | 3 | GO:0006519 | 63 | 2.40% | 9.19E-04 | 49 | 2.09 | 4.88E-02 |
| cellular protein catabolism | 5 | GO:0044265 | 38 | 1.45% | 1.60E-02 | 35 | 1.49 | 1.60E-02 |
| cellular protein metabolism | 4 | GO:0044260 | 505 | 19.25% | 5.72E-03 | 497 | 21.18 | 8.08E-07 |
| establishment of protein localization | 2, 3, 4 | GO:0045184 | 133 | 5.07% | 5.37E-09 | 123 | 5.24 | 5.49E-09 |
| intracellular protein transport | 4, 5 | GO:0006886 | 76 | 2.90% | 6.68E-06 | 75 | 3.2 | 2.54E-07 |
| modification-dependent protein catabolism | >5 | GO:0019941 | 31 | 1.18% | 2.43E-02 | 30 | 1.28 | 1.11E-02 |
| negative regulation of enzyme activity | 4 | GO:0043086 | 16 | 0.61% | 9.59E-03 | 13 | 0.55 | 4.76E-02 |
| negative regulation of protein kinase activity | >5 | GO:0006469 | 13 | 0.50% | 3.25E-03 | 10 | 0.43 | 3.57E-02 |
| negative regulation of transferase activity | 5 | GO:0051348 | 13 | 0.50% | 3.25E-03 | 10 | 0.43 | 3.57E-02 |
| protein catabolism | 4, 5 | GO:0030163 | 48 | 1.83% | 3.93E-03 | 45 | 1.92 | 2.63E-03 |
| protein complex assembly | 4, 5 | GO:0006461 | 70 | 2.67% | 2.49E-06 | 56 | 2.39 | 8.05E-04 |
| protein import | 4, 5 | GO:0017038 | 21 | 0.80% | 2.17E-03 | 19 | 0.81 | 3.87E-03 |
| protein import into nucleus | 5 | GO:0006606 | 20 | 0.76% | 5.32E-04 | 18 | 0.77 | 1.24E-03 |
| protein localization | 3 | GO:0008104 | 140 | 5.34% | 4.63E-10 | 128 | 5.45 | 1.38E-09 |
| protein metabolism | 3 | GO:0019538 | 570 | 21.72% | 3.75E-05 | 548 | 23.35 | 8.13E-09 |
| protein modification | 5 | GO:0006464 | 289 | 11.01% | 1.21E-04 | 249 | 10.61 | 4.94E-03 |
| protein polymerization | 5 | GO:0051258 | 17 | 0.65% | 1.08E-03 | 13 | 0.55 | 2.15E-02 |
| protein targeting | 5 | GO:0006605 | 37 | 1.41% | 3.61E-03 | 35 | 1.49 | 2.23E-03 |
| protein transport | 3, 4, 5 | GO:0015031 | 128 | 4.88% | 1.51E-08 | 118 | 5.03 | 1.91E-08 |
| proteolysis during cellular protein catabolism | >5 | GO:0051603 | 38 | 1.45% | 1.47E-02 | 35 | 1.49 | 1.48E-02 |
| regulation of enzyme activity | 3 | GO:0050790 | 56 | 2.13% | 3.81E-04 | 44 | 1.87 | 2.15E-02 |
| regulation of protein metabolism | 3, 4 | GO:0051246 | 52 | 1.98% | 9.12E-05 | 44 | 1.87 | 1.45E-03 |
| regulation of transferase activity | 4 | GO:0051338 | 32 | 1.22% | 3.75E-03 | 26 | 1.11 | 3.36E-02 |
| regulation of translation | 3, 5 | GO:0006417 | 19 | 0.72% | 3.47E-02 | 21 | 0.89 | 2.71E-03 |
| ubiquitin cycle | >5 | GO:0006512 | 89 | 3.39% | 2.64E-02 | 81 | 3.45 | 2.79E-02 |
| ubiquitin-dependent protein catabolism | >5 | GO:0006511 | 31 | 1.18% | 2.43E-02 | 30 | 1.28 | 1.11E-02 |
|  |  |  |  |  |  |  |  |  |
| **Signal transduction/Pathways** |  |  |  |  |  |  |  |  |
| I-kappaB kinase/NF-kappaB cascade | >5 | GO:0007249 | 34 | 1.30% | 3.89E-05 | 28 | 1.19 | 1.10E-03 |
| intracellular signaling cascade | 4 | GO:0007242 | 215 | 8.19% | 7.91E-07 | 175 | 7.46 | 2.04E-03 |
| positive regulation of I-kappaB kinase/NF-kappaB cascade | 5 | GO:0043123 | 28 | 1.07% | 5.46E-06 | 24 | 1.02 | 8.54E-05 |
| positive regulation of signal transduction | 4, 5 | GO:0009967 | 33 | 1.26% | 1.74E-06 | 27 | 1.15 | 1.33E-04 |
| protein kinase cascade | 5 | GO:0007243 | 68 | 2.59% | 7.76E-06 | 50 | 2.13 | 1.30E-02 |
| regulation of I-kappaB kinase/NF-kappaB cascade | 4, 5 | GO:0043122 | 30 | 1.14% | 2.53E-06 | 25 | 1.07 | 1.02E-04 |
| regulation of signal transduction | 3, 4 | GO:0009966 | 61 | 2.32% | 4.03E-07 | 50 | 2.13 | 8.56E-05 |
| secretory pathway | 3, 4, 5 | GO:0045045 | 52 | 1.98% | 2.11E-06 | 39 | 1.66 | 2.46E-03 |
| small GTPase mediated signal transduction | 5 | GO:0007264 | 55 | 2.10% | 8.46E-03 | 50 | 2.13 | 1.08E-02 |
|  |  |  |  |  |  |  |  |  |
| **Others** |  |  |  |  |  |  |  |  |
| alcohol biosynthesis | 4 | GO:0046165 | 12 | 0.46% | 7.84E-03 | 11 | 0.47 | 1.10E-02 |
| biopolymer catabolism | 4 | GO:0043285 | 51 | 1.94% | 3.09E-03 | 45 | 1.92 | 8.56E-03 |
| biopolymer metabolism | 3 | GO:0043283 | 518 | 19.74% | 5.53E-11 | 456 | 19.43 | 2.81E-08 |
| biopolymer modification | 4 | GO:0043412 | 301 | 11.47% | 3.30E-05 | 258 | 10.99 | 2.87E-03 |
| catabolism | 2 | GO:0009056 | 120 | 4.57% | 5.12E-04 | 125 | 5.33 | 3.86E-07 |
| cell migration | 3, 4 | GO:0016477 | 31 | 1.18% | 5.44E-05 | 24 | 1.02 | 4.39E-03 |
| cell motility | 2, 3 | GO:0006928 | 64 | 2.44% | 2.72E-07 | 54 | 2.3 | 2.33E-05 |
| cell organization and biogenesis | 2 | GO:0016043 | 324 | 12.35% | 8.42E-16 | 296 | 12.61 | 3.99E-15 |
| cell proliferation | 2 | GO:0008283 | 131 | 4.99% | 1.66E-10 | 109 | 4.64 | 6.20E-07 |
| cellular catabolism | 3 | GO:0044248 | 100 | 3.81% | 1.87E-03 | 104 | 4.43 | 5.65E-06 |
| cellular localization | 2, 3 | GO:0051641 | 140 | 5.34% | 5.03E-09 | 129 | 5.5 | 6.58E-09 |
| cellular macromolecule catabolism | 4 | GO:0044265 | 68 | 2.59% | 5.57E-03 | 59 | 2.51 | 2.13E-02 |
| cellular macromolecule metabolism | 3 | GO:0044260 | 520 | 19.82% | 1.59E-03 | 506 | 21.56 | 4.10E-07 |
| cellular metabolism | 2 | GO:0044237 | 1207 | 46.00% | 1.26E-05 | 1132 | 48.23 | 3.23E-10 |
| cellular morphogenesis | 4, 5 | GO:0000902 | 56 | 2.13% | 3.99E-03 | 51 | 2.17 | 5.19E-03 |
| cellular physiological process | 1 | GO:0009987 | 1690 | 64.41% | 8.74E-18 | 1535 | 65.4 | 1.17E-18 |
| cellular process | 1 | GO:0009987 | 1903 | 72.52% | 2.54E-07 | 1710 | 72.86 | 7.35E-06 |
| detection of pest, pathogen or parasite |  | no GO ID | 5 | 0.19% | 3.91E-02 | 5 | 0.21 | 2.77E-02 |
| development | 1 | GO:0032502 | 336 | 12.80% | 9.02E-08 | 275 | 11.72 | 1.69E-03 |
| ER to Golgi vesicle-mediated transport | 4, 5 | GO:0006888 | 23 | 0.88% | 3.76E-05 | 18 | 0.77 | 2.18E-03 |
| establishment of cellular localization | 2, 3, 4 | GO:0051649 | 136 | 5.18% | 3.31E-08 | 126 | 5.37 | 2.60E-08 |
| establishment of localization | 1, 2 | GO:0051234 | 476 | 18.14% | 3.84E-03 | 431 | 18.36 | 4.17E-03 |
| Golgi vesicle transport | 4, 5 | GO:0048193 | 32 | 1.22% | 4.18E-06 | 25 | 1.07 | 7.22E-04 |
| intracellular transport | 3, 4, 5 | GO:0046907 | 134 | 5.11% | 4.75E-08 | 124 | 5.28 | 4.07E-08 |
| localization | 1 | GO:0051179 | 485 | 18.48% | 1.03E-03 | 437 | 18.62 | 1.99E-03 |
| localization of cell | 2 | GO:0051674 | 64 | 2.44% | 2.72E-07 | 54 | 2.3 | 2.33E-05 |
| locomotion | 1 | GO:0040011 | 64 | 2.44% | 2.72E-07 | 54 | 2.3 | 2.33E-05 |
| macromolecule catabolism | 3 | GO:0009057 | 78 | 2.97% | 1.56E-03 | 69 | 2.94 | 4.86E-03 |
| macromolecule metabolism | 2 | GO:0043170 | 803 | 30.60% | 7.08E-12 | 765 | 32.59 | 3.57E-18 |
| maintenance of localization | 1 | GO:0051235 | 11 | 0.42% | 5.63E-03 | 12 | 0.51 | 6.52E-04 |
| metabolism | 1 | GO:0008152 | 1304 | 49.70% | 2.80E-07 | 1213 | 51.68 | 1.65E-11 |
| morphogenesis | 2, 3 | GO:0009653 | 115 | 4.38% | 3.55E-04 | 91 | 3.88 | 4.53E-02 |
| muscle development | 4, 5 | GO:0007517 | 23 | 0.88% | 2.02E-02 | 23 | 0.98 | 6.35E-03 |
| negative regulation of biological process | 3 | GO:0048519 | 194 | 7.39% | 1.88E-17 | 167 | 7.12 | 7.14E-13 |
| negative regulation of cell organization and biogenesis | 4, 5 | GO:0051129 | 13 | 0.50% | 5.51E-04 | 9 | 0.38 | 3.35E-02 |
| negative regulation of cell proliferation | 4, 5 | GO:0008285 | 42 | 1.60% | 2.59E-05 | 36 | 1.53 | 3.44E-04 |
| negative regulation of cellular metabolism | 4, 5 | GO:0031324 | 47 | 1.79% | 7.35E-04 | 40 | 1.7 | 5.79E-03 |
| negative regulation of cellular physiological process | 3, 4 | GO:0048523 | 169 | 6.44% | 7.18E-17 | 145 | 6.18 | 2.09E-12 |
| negative regulation of cellular process | 3, 4 | GO:0048523 | 183 | 6.97% | 3.31E-17 | 158 | 6.73 | 6.92E-13 |
| negative regulation of metabolism | 3, 4 | GO:0009892 | 60 | 2.29% | 8.24E-06 | 48 | 2.05 | 1.40E-03 |
| negative regulation of physiological process | 3 | GO:0048519 | 170 | 6.48% | 2.18E-15 | 146 | 6.22 | 1.79E-11 |
| nitrogen compound metabolism | 2 | GO:0006807 | 82 | 3.12% | 2.41E-04 | 65 | 2.77 | 2.18E-02 |
| nuclear export | >5 | GO:0051168 | 15 | 0.57% | 6.82E-03 | 14 | 0.6 | 7.31E-03 |
| nuclear import | >5 | GO:0051170 | 21 | 0.80% | 2.27E-04 | 19 | 0.81 | 5.29E-04 |
| nuclear transport | 4, 5 | GO:0051169 | 35 | 1.33% | 3.39E-06 | 31 | 1.32 | 2.38E-05 |
| nucleocytoplasmic transport | 5 | GO:0006913 | 37 | 1.41% | 1.99E-06 | 35 | 1.49 | 1.48E-06 |
| organ development | 3, 4 | GO:0048513 | 111 | 4.23% | 1.59E-05 | 84 | 3.58 | 2.67E-02 |
| organelle organization and biogenesis | 3 | GO:0006996 | 167 | 6.36% | 2.62E-06 | 141 | 6.01 | 4.21E-04 |
| organic acid metabolism | 3 | GO:0006082 | 104 | 3.96% | 2.83E-05 | 104 | 4.43 | 2.46E-07 |
| physiological process | 1 | GO:0008150 | 1842 | 70.20% | 3.96E-04 | 1673 | 71.28 | 1.70E-05 |
| plasma membrane organization and biogenesis | 4 | GO:0007009 | 5 | 0.19% | 1.64E-02 | 5 | 0.21 | 1.14E-02 |
| positive regulation of biological process | 3 | GO:0048518 | 158 | 6.02% | 1.09E-13 | 127 | 5.41 | 7.87E-08 |
| positive regulation of cell proliferation | 4, 5 | GO:0008284 | 34 | 1.30% | 9.46E-04 | 32 | 1.36 | 7.03E-04 |
| positive regulation of cellular physiological process | 3, 4 | GO:0048522 | 109 | 4.15% | 1.18E-08 | 92 | 3.92 | 4.23E-06 |
| positive regulation of cellular process | 3, 4 | GO:0048522 | 136 | 5.18% | 3.69E-12 | 116 | 4.94 | 6.91E-09 |
| positive regulation of physiological process | 3 | GO:0048518 | 115 | 4.38% | 2.02E-09 | 96 | 4.09 | 2.35E-06 |
| primary metabolism | 2 | GO:0044238 | 1199 | 45.69% | 9.09E-09 | 1097 | 46.74 | 4.92E-10 |
| pyruvate metabolism | >5 | GO:0006090 | 12 | 0.46% | 2.85E-03 | 10 | 0.43 | 1.43E-02 |
| regulation of biological process | 2 | GO:0050789 | 649 | 24.73% | 1.68E-10 | 531 | 22.62 | 1.69E-03 |
| regulation of body fluids | 2, 3 | GO:0050878 | 23 | 0.88% | 4.43E-02 | 22 | 0.94 | 2.87E-02 |
| regulation of cell organization and biogenesis | 3, 4 | GO:0051128 | 26 | 0.99% | 2.36E-08 | 16 | 0.68 | 3.02E-03 |
| regulation of cell proliferation | 3, 4 | GO:0042127 | 78 | 2.97% | 5.91E-08 | 70 | 2.98 | 4.46E-07 |
| regulation of cellular physiological process | 2, 3 | GO:0050794 | 569 | 21.68% | 8.33E-08 | 466 | 19.86 | 1.25E-02 |
| regulation of cellular process | 2, 3 | GO:0050794 | 605 | 23.06% | 2.19E-09 | 496 | 21.13 | 3.01E-03 |
| regulation of physiological process | 2 | GO:0050789 | 584 | 22.26% | 8.50E-08 | 477 | 20.32 | 1.82E-02 |
| response to other organism | 2, 3 | GO:0051707 | 133 | 5.07% | 5.65E-08 | 99 | 4.22 | 4.62E-03 |
| response to pest, pathogen or parasite |  | no GO ID | 132 | 5.03% | 2.06E-09 | 99 | 4.22 | 6.59E-04 |
| response to stress | 2 | GO:0006950 | 234 | 8.92% | 1.86E-11 | 191 | 8.14 | 3.30E-06 |
| response to virus | 3, 4 | GO:0009615 | 25 | 0.95% | 1.87E-04 | 18 | 0.77 | 2.16E-02 |
| response to wounding | 3 | GO:0009611 | 83 | 3.16% | 1.73E-04 | 63 | 2.68 | 4.54E-02 |
| secretion | 2, 3 | GO:0046903 | 60 | 2.29% | 7.19E-06 | 48 | 2.05 | 1.28E-03 |
| vesicle-mediated transport | 3, 4 | GO:0016192 | 89 | 3.39% | 1.57E-07 | 59 | 2.51 | 3.96E-02 |
