# Supplementary material for: Improved Elucidation of Biological Processes Linked to Diabetic Nephropathy by Single Probe-Based Microarray Data Analysis
Source: PLoS One. 2008 Aug 13;3(8):e2937. doi: 10.1371/journal.pone.0002937 (PMC2493035; doi:10.1371/journal.pone.0002937)
Supplement: Table S3 — DAVID analysis of all regulated genes and transcripts found by the two independent array analysis techniques. Shown are the prominent biological aspects found by CI only (“Class 1”) or RMA only (“Class 2”). In total 174 common GO-categories were found (see, Table S2), additional 104 GO-categories were uniquely associated with the CI-derived gene list, while 63 additional GO-categories were found by RMA only. (0.35 MB DOC) [file pone.0002937.s003.doc]

**Table S3: DAVID analysis of all regulated genes and transcripts found by the two independent array analysis techniques**

Shown are the prominent biological aspects found by CI only (“Class 1”) or RMA only (“Class 2”). In total 174 common GO-categories were found (see, Table S2), additional 104 GO-categories were uniquely associated with the CI-derived gene list, while 63 additional GO-categories were found by RMA only.

| **GO categories CI unique (“Class 1”)** | | | | | |  | **GO categories RMA/SAM unique (“Class 2”)** | | | | | | | | | |
| --- | --- | --- | --- | --- | --- | --- | --- | --- | --- | --- | --- | --- | --- | --- | --- | --- |
|  | **GO ID** | **GO Level** | **Genes involved in the term** | **involved genes/total genes**  **(%)** | **p-value (Ease-Score DAVID)** |  |  | **GO ID** | | **GO Level** | **Genes involved in the term** | | **involved genes/**  **total genes**  **(%)** | | **p-value (Ease-Score DAVID)** | |
| **Angiogenesis** |  |  |  |  |  |  |  |  | |  |  | |  | |  | |
| angiogenesis | GO:0001525 | 3, 4, 5 | 19 | 0.72% | 5.31E-03 |  |  |  | |  |  | |  | |  | |
| blood vessel development | GO:0001568 | 5 | 20 | 0.76% | 3.71E-03 |  |  |  | |  |  | |  | |  | |
| blood vessel morphogenesis | GO:0048514 | 4, 5 | 20 | 0.76% | 3.71E-03 |  |  |  | |  |  | |  | |  | |
| vasculature development | GO:0001944 | 4, 5 | 20 | 0.76% | 3.71E-03 |  |  |  | |  |  | |  | |  | |
|  |  |  |  |  |  |  |  |  | |  |  | |  | |  | |
| **Carbohydrates** |  |  |  |  |  |  | **Carbohydrates** |  | |  |  | |  | |  | |
| glucose metabolism | GO:0006006 | >5 | 25 | 0.95% | 4.43E-02 |  | carbohydrate metabolism | GO:0005975 | | 3 | 88 | | 3.75% | | 4.44E-03 | |
| glucose transport | GO:0015758 | >5 | 7 | 0.27% | 2.27E-02 |  | cellular carbohydrate metabolism | GO:0044262 | | 4 | 63 | | 2.68% | | 5.50E-03 | |
| hexose transport | GO:0008645 | 5 | 8 | 0.30% | 7.81E-03 |  | hexose metabolism | GO:0019318 | | 5 | 31 | | 1.32% | | 2.26E-02 | |
| monosaccharide transport | GO:0015749 | 4, 5 | 8 | 0.30% | 7.81E-03 |  | monosaccharide metabolism | GO:0005996 | | 4, 5 | 32 | | 1.36% | | 1.84E-02 | |
|  |  |  |  |  |  |  |  |  | |  |  | |  | |  | |
| **Cell Cycle** |  |  |  |  |  |  | **Cell Cycle** |  | |  |  | |  | |  | |
| cell cycle arrest | GO:0007050 | 5 | 16 | 0.61% | 4.46E-02 |  | cell division | GO:0051301 | | 2 | 32 | | 1.36% | | 3.80E-02 | |
|  |  |  |  |  |  |  | M phase | GO:0000279 | | 4, 5 | 35 | | 1.49% | | 3.24E-02 | |
|  |  |  |  |  |  |  | M phase of mitotic cell cycle | GO:0000087 | | 4, 5 | 33 | | 1.41% | | 2.31E-03 | |
|  |  |  |  |  |  |  | mitosis | GO:0007067 | | 4, 5 | 33 | | 1.41% | | 1.85E-03 | |
|  |  |  |  |  |  |  |  |  | |  |  | |  | |  | |
| **Cytosceleton** |  |  |  |  |  |  |  |  | |  |  | |  | |  | |
| actin filament capping | GO:0051693 |  | 9 | 0.34% | 7.22E-03 |  |  |  | |  |  | |  | |  | |
| actin filament depolymerization | GO:0030042 | 5 | 9 | 0.34% | 7.22E-03 |  |  |  | |  |  | |  | |  | |
| barbed-end actin filament capping | GO:0051016 | >5 | 9 | 0.34% | 7.22E-03 |  |  |  | |  |  | |  | |  | |
| negative regulation of actin filament depolymerization | GO:0030835 | 5 | 9 | 0.34% | 7.22E-03 |  |  |  | |  |  | |  | |  | |
| positive regulation of actin filament polymerization | GO:0030838 | 5 | 4 | 0.15% | 2.16E-02 |  |  |  | |  |  | |  | |  | |
| regulation of actin filament depolymerization | GO:0030834 | 5 | 9 | 0.34% | 7.22E-03 |  |  |  | |  |  | |  | |  | |
| regulation of actin filament polymerization | GO:0030833 | 5 | 8 | 0.30% | 7.94E-04 |  |  |  | |  |  | |  | |  | |
|  |  |  |  |  |  |  |  |  | |  |  | |  | |  | |
| **DNA/RNA** |  |  |  |  |  |  | **DNA/RNA** |  | |  |  | |  | |  | |
| DNA metabolism | GO:0006259 | 4 | 112 | 4.27% | 3.79E-02 |  | DNA ligation | GO:0006266 | | 5 | 4 | | 0.17% | | 2.94E-02 | |
| DNA packaging | GO:0006323 | 5 | 46 | 1.75% | 4.77E-02 |  | mRNA catabolism | GO:0006402 | |  | 7 | | 0.30% | | 4.88E-02 | |
| double-strand break repair | GO:0006302 | 5 | 7 | 0.27% | 4.93E-02 |  | regulation of mRNA stability | GO:0043488 | | 4 | 3 | | 0.13% | | 4.32E-02 | |
| nucleobase, nucleoside, nucleotide and nucleic acid metabolism | GO:0006139 | 3 | 554 | 21.11% | 3.23E-03 |  | regulation of RNA stability | GO:0043487 | | 3 | 3 | | 0.13% | | 4.32E-02 | |
| nucleoside monophosphate biosynthesis | GO:0009124 | 5 | 8 | 0.30% | 1.96E-02 |  | viral genome replication | GO:0019079 | | 3, 4, 5 | 8 | | 0.34% | | 1.13E-02 | |
| nucleoside monophosphate metabolism | GO:0009123 |  | 8 | 0.30% | 1.96E-02 |  |  |  | |  |  | |  | |  | |
| positive regulation of nucleobase, nucleoside, nucleotide and nucleic acid metabolism | GO:0045935 | 5 | 26 | 0.99% | 3.73E-02 |  |  |  | |  |  | |  | |  | |
| purine nucleoside monophosphate biosynthesis | GO:0009127 | >5 | 6 | 0.23% | 4.67E-02 |  |  |  | |  |  | |  | |  | |
| purine nucleoside monophosphate metabolism | GO:0009126 | >5 | 6 | 0.23% | 4.67E-02 |  |  |  | |  |  | |  | |  | |
| purine ribonucleoside monophosphate biosynthesis | GO:0009168 | >5 | 6 | 0.23% | 4.67E-02 |  |  |  | |  |  | |  | |  | |
| purine ribonucleoside monophosphate metabolism | GO:0009167 | >5 | 6 | 0.23% | 4.67E-02 |  |  |  | |  |  | |  | |  | |
| regulation of DNA metabolism | GO:0051052 | 5 | 12 | 0.46% | 4.86E-03 |  |  |  | |  |  | |  | |  | |
| regulation of DNA replication | GO:0006275 | >5 | 6 | 0.23% | 4.67E-02 |  |  |  | |  |  | |  | |  | |
| ribonucleoside monophosphate biosynthesis | GO:0009156 | >5 | 8 | 0.30% | 1.48E-02 |  |  |  | |  |  | |  | |  | |
| ribonucleoside monophosphate metabolism | GO:0009161 | >5 | 8 | 0.30% | 1.48E-02 |  |  |  | |  |  | |  | |  | |
|  |  |  |  |  |  |  |  |  | |  |  | |  | |  | |
| **Immune response/Inflammation** |  |  |  |  |  |  |  |  | |  |  | |  | |  | |
| antimicrobial humoral response | GO:0019730 | 3, 4 | 21 | 0.80% | 2.37E-02 |  |  |  | |  |  | |  | |  | |
| antimicrobial humoral response (sensu Vertebrata) | GO:0019730 | 3, 4 | 20 | 0.76% | 2.87E-02 |  |  |  | |  |  | |  | |  | |
| defense response | GO:0006952 | 2 | 211 | 8.04% | 3.44E-03 |  |  |  | |  |  | |  | |  | |
| immune cell activation | GO:0045321 | 2, 3 | 25 | 0.95% | 1.72E-02 |  |  |  | |  |  | |  | |  | |
| immune response | GO:0006955 | 2 | 198 | 7.55% | 9.01E-04 |  |  |  | |  |  | |  | |  | |
| inflammatory response | GO:0006954 | 3, 4 | 47 | 1.79% | 1.53E-03 |  |  |  | |  |  | |  | |  | |
| lymphocyte activation | GO:0046649 | 3, 4 | 23 | 0.88% | 7.79E-03 |  |  |  | |  |  | |  | |  | |
| positive regulation of immune response | GO:0050778 | 4, 5 | 14 | 0.53% | 3.58E-02 |  |  |  | |  |  | |  | |  | |
| positive regulation of lymphocyte activation | GO:0051251 | 5 | 10 | 0.38% | 6.44E-03 |  |  |  | |  |  | |  | |  | |
| positive regulation of T cell activation | GO:0050870 | >5 | 8 | 0.30% | 3.22E-02 |  |  |  | |  |  | |  | |  | |
| regulation of lymphocyte activation | GO:0051249 | 4, 5 | 13 | 0.50% | 9.99E-03 |  |  |  | |  |  | |  | |  | |
| regulation of T cell activation | GO:0050863 | 5 | 10 | 0.38% | 4.60E-02 |  |  |  | |  |  | |  | |  | |
| response to external stimulus | GO:0009605 | 2 | 98 | 3.73% | 1.61E-03 |  |  |  | |  |  | |  | |  | |
| T cell activation | GO:0042110 | 4, 5 | 14 | 0.53% | 2.33E-02 |  |  |  | |  |  | |  | |  | |
|  |  |  |  |  |  |  |  |  | |  |  | |  | |  | |
| **Lipids/Fatty acids** |  |  |  |  |  |  | **Lipids/Fatty acids** |  | |  |  | |  | |  | |
| membrane lipid metabolism | GO:0006643 | 5 | 28 | 1.07% | 2.64E-02 |  | cellular lipid metabolism | GO:0044255 | | 3, 4 | 85 | | 3.62% | | 2.45E-03 | |
| sphingolipid metabolism | GO:0006665 | 5 | 13 | 0.50% | 2.86E-02 |  | fatty acid beta-oxidation | GO:0006635 | |  | 9 | | 0.38% | | 1.26E-03 | |
|  |  |  |  |  |  |  | fatty acid metabolism | GO:0006631 | | 4, 5 | 40 | | 1.70% | | 5.74E-05 | |
|  |  |  |  |  |  |  | fatty acid oxidation | GO:0019395 | | 5 | 12 | | 0.51% | | 2.75E-03 | |
|  |  |  |  |  |  |  | glycerophospholipid metabolism | GO:0006650 | | >5 | 11 | | 0.47% | | 2.71E-02 | |
|  |  |  |  |  |  |  | lipid catabolism | GO:0016042 | | 3, 4 | 18 | | 0.77% | | 4.91E-02 | |
|  |  |  |  |  |  |  | lipid metabolism | GO:0006629 | | 3 | 106 | | 4.52% | | 1.29E-03 | |
|  |  |  |  |  |  |  |  |  | |  |  | |  | |  | |
| **Neuron Development** |  |  |  |  |  |  | **Neuron Development** |  | | |  |  | |  | |  |
| regulation of neurogenesis | GO:0050767 | 3, 4, 5 | 7 | 0.27% | 1.17E-02 |  | dendrite morphogenesis | GO:0048813 | | 4 | 4 | | 0.17% | | 1.62E-02 | |
|  |  |  |  |  |  |  | neurite morphogenesis | GO:0048812 | | 5 | 15 | | 0.64% | | 1.47E-02 | |
|  |  |  |  |  |  |  | neurogenesis | GO:0022008 | | 3, 5 | 24 | | 1.02% | | 4.97E-03 | |
|  |  |  |  |  |  |  | neuron development | GO:0048666 | | 3, 5 | 20 | | 0.85% | | 7.09E-03 | |
|  |  |  |  |  |  |  | neuron differentiation | GO:0030182 | | 4, 5 | 22 | | 0.94% | | 1.15E-02 | |
|  |  |  |  |  |  |  | neuron morphogenesis during differentiation | GO:0048667 | | 4 | 15 | | 0.64% | | 1.47E-02 | |
|  |  |  |  |  |  |  |  |  | |  |  | |  | |  | |
| **Proteins/Enzymes** |  |  |  |  |  |  | **Proteins/Enzymes** |  | |  |  | |  | |  | |
| amino acid metabolism | GO:0006520 | 4, 5 | 53 | 2.02% | 4.00E-03 |  | amino acid catabolism | GO:0009063 | | 5 | 15 | | 0.64% | | 2.52E-02 | |
| negative regulation of protein metabolism | GO:0051248 | 4, 5 | 17 | 0.65% | 6.64E-03 |  | glycoprotein metabolism | GO:0009100 | | 5 | 28 | | 1.19% | | 3.05E-02 | |
| peptidyl-amino acid modification | GO:0018193 | >5 | 16 | 0.61% | 3.82E-03 |  | protein amino acid N-linked glycosylation | GO:0006487 | | >5 | 11 | | 0.47% | | 2.71E-02 | |
| peptidyl-tyrosine modification | GO:0018212 | >5 | 9 | 0.34% | 1.67E-02 |  | protein biosynthesis (translation) | GO:0006412 | | 2, 4, 5 | 141 | | 6.01% | | 6.12E-05 | |
| peptidyl-tyrosine phosphorylation | GO:0018108 | >5 | 8 | 0.30% | 3.22E-02 |  | protein folding | GO:0006457 | | 5 | 50 | | 2.13% | | 1.15E-02 | |
| positive regulation of enzyme activity | GO:0043085 | 4 | 29 | 1.11% | 9.04E-04 |  | protein retention in ER | GO:0006621 | | 3, 4, 5 | 4 | | 0.17% | | 4.66E-02 | |
| positive regulation of protein kinase activity | GO:0045860 | >5 | 15 | 0.57% | 2.92E-02 |  | protein targeting to membrane | GO:0006612 | |  | 9 | | 0.38% | | 2.70E-03 | |
| positive regulation of transferase activity | GO:0051347 | 5 | 16 | 0.61% | 1.55E-02 |  | regulation of translational initiation | GO:0006446 | | 4, 5 | 11 | | 0.47% | | 5.67E-03 | |
| protein amino acid phosphorylation | GO:0006468 | >5 | 106 | 4.04% | 4.03E-02 |  | translation | GO:0006412 | | 2, 4, 5 | 33 | | 1.41% | | 4.37E-02 | |
| protein depolymerization | GO:0051261 | 5 | 13 | 0.50% | 5.51E-04 |  | translational initiation | GO:0006413 | | 3, 5 | 15 | | 0.64% | | 1.47E-02 | |
| regulation of amino acid metabolism | GO:0006521 | 5 | 8 | 0.30% | 1.96E-02 |  |  |  | |  |  | |  | |  | |
| regulation of kinase activity | GO:0043549 | 5 | 31 | 1.18% | 6.32E-03 |  |  |  | |  |  | |  | |  | |
| regulation of MAPK activity | GO:0043405 | >5 | 16 | 0.61% | 6.77E-03 |  |  |  | |  |  | |  | |  | |
| regulation of protein amino acid phosphorylation | GO:0001932 | >5 | 7 | 0.27% | 4.93E-02 |  |  |  | |  |  | |  | |  | |
| regulation of protein kinase activity | GO:0045859 | >5 | 31 | 1.18% | 6.32E-03 |  |  |  | |  |  | |  | |  | |
|  |  |  |  |  |  |  |  |  | |  |  | |  | |  | |
| **Signal transduction/Pathways** | |  |  |  |  |  | **Signal transduction/Pathways** | | |  |  | |  | |  | |
| enzyme linked receptor protein signaling pathway | GO:0007167 | 5 | 44 | 1.68% | 5.76E-04 |  | negative regulation of signal transduction | | GO:0009968 | 4, 5 | 14 | | 0.60% | | 4.15E-02 | |
| Notch signaling pathway | GO:0007219 | 5 | 11 | 0.42% | 1.80E-02 |  |  | |  |  |  | |  | |  | |
| Rho protein signal transduction | GO:0007266 |  | 9 | 0.34% | 1.67E-02 |  |  | |  |  |  | |  | |  | |
| signal transduction | GO:0007165 | 3 | 488 | 18.60% | 2.48E-02 |  |  | |  |  |  | |  | |  | |
| transmembrane receptor protein tyrosine kinase signaling pathway | GO:0007169 |  | 32 | 1.22% | 2.96E-03 |  |  | |  |  |  | |  | |  | |
| Wnt receptor signaling pathway | GO:0016055 | 5 | 23 | 0.88% | 2.25E-02 |  |  | |  |  |  | |  | |  | |
|  |  |  |  |  |  |  |  | |  |  |  | |  | |  | |
| **Others** |  |  |  |  |  |  | **Others** | |  |  |  | |  | |  | |
| amine metabolism | GO:0009308 | 3 | 74 | 2.82% | 1.38E-03 |  | aerobic respiration | | GO:0009060 | 5 | 11 | | 0.47% | | 8.90E-03 | |
| cell activation | GO:0001775 | 2 | 25 | 0.95% | 1.91E-02 |  | alcohol metabolism | | GO:0006066 | 3 | 56 | | 2.39% | | 1.43E-03 | |
| cell adhesion | GO:0007155 | 2 | 132 | 5.03% | 6.68E-05 |  | amine catabolism | | GO:0009310 | 4, 5 | 16 | | 0.68% | | 1.72E-02 | |
| cell differentiation | GO:0030154 | 3 | 91 | 3.47% | 1.03E-02 |  | biosynthesis | | GO:0009058 | 2 | 250 | | 10.65% | | 8.51E-07 | |
| cell fate commitment | GO:0045165 | 2 | 7 | 0.27% | 4.93E-02 |  | blood coagulation | | GO:0007596 | 3, 4, 5 | 19 | | 0.81% | | 3.55E-02 | |
| di-, tri-valent inorganic cation homeostasis | GO:0055066 | >5 | 23 | 0.88% | 4.84E-02 |  | cell development | | GO:0048468 | 2, 4 | 23 | | 0.98% | | 3.89E-02 | |
| embryonic development | GO:0009790 | 2, 3 | 21 | 0.80% | 5.73E-03 |  | cell-matrix adhesion | | GO:0007160 | 4 | 15 | | 0.64% | | 2.86E-02 | |
| embryonic pattern specification | GO:0009880 | 3 | 5 | 0.19% | 3.91E-02 |  | cell-substrate adhesion | | GO:0031589 | 3 | 15 | | 0.64% | | 2.86E-02 | |
| endocytosis | GO:0006897 | 4, 5 | 35 | 1.33% | 2.41E-03 |  | cellular biosynthesis | | GO:0044249 | 3 | 221 | | 9.42% | | 1.40E-05 | |
| endosome transport | GO:0016197 | 4, 5 | 9 | 0.34% | 1.29E-02 |  | cellular morphogenesis during differentiation | | GO:0000904 | 3, 5 | 16 | | 0.68% | | 1.31E-02 | |
| hemopoiesis | GO:0030097 | 4, 5 | 23 | 0.88% | 5.95E-03 |  | cellular respiration | | GO:0045333 | 4 | 11 | | 0.47% | | 1.62E-02 | |
| hemopoietic or lymphoid organ development | GO:0048534 | 3, 4, 5 | 23 | 0.88% | 6.82E-03 |  | coagulation | | GO:0050817 | 2 | 20 | | 0.85% | | 2.30E-02 | |
| membrane organization and biogenesis | GO:0016044 | 3 | 16 | 0.61% | 4.65E-03 |  | coenzyme catabolism | | GO:0009109 | 5 | 9 | | 0.38% | | 2.79E-02 | |
| nitric oxide biosynthesis | GO:0006809 | 4, 5 | 8 | 0.30% | 7.81E-03 |  | cytoplasm organization and biogenesis | | GO:0007028 | 3 | 23 | | 0.98% | | 2.91E-03 | |
| nitric oxide metabolism | GO:0046209 | 3 | 8 | 0.30% | 7.81E-03 |  | electron transport | | GO:0006118 | 3 | 87 | | 3.71% | | 6.37E-05 | |
| NLS-bearing substrate import into nucleus | GO:0006607 |  | 6 | 0.23% | 1.76E-02 |  | generation of precursor metabolites and energy | | GO:0006091 | 2 | 131 | | 5.58% | | 1.22E-05 | |
| organ morphogenesis | GO:0009887 | 3, 4, 5 | 43 | 1.64% | 1.80E-02 |  | glutamate biosynthesis | | GO:0006537 |  | 3 | | 0.13% | | 4.32E-02 | |
| positive regulation of cell activation | GO:0050867 |  | 11 | 0.42% | 3.20E-03 |  | intercellular junction assembly | | GO:0007043 | 5 | 6 | | 0.26% | | 2.33E-02 | |
| positive regulation of cellular metabolism | GO:0031325 | 4, 5 | 33 | 1.26% | 1.89E-02 |  | intercellular junction assembly and maintenance | | GO:0045216 | 4 | 8 | | 0.34% | | 2.98E-03 | |
| positive regulation of metabolism | GO:0009893 | 3, 4 | 41 | 1.56% | 7.02E-04 |  | lamellipodium biogenesis | | GO:0030032 | >5 | 3 | | 0.13% | | 4.32E-02 | |
| positive regulation of nitric oxide biosynthesis | GO:0045429 | 5 | 4 | 0.15% | 2.16E-02 |  | macromolecule biosynthesis | | GO:0009059 | 3 | 162 | | 6.90% | | 1.86E-06 | |
| positive regulation of organismal physiological process | GO:0051240 | 3, 4 | 15 | 0.57% | 2.92E-02 |  | negative regulation of cell adhesion | | GO:0007162 | 4, 5 | 6 | | 0.26% | | 4.11E-02 | |
| receptor mediated endocytosis | GO:0006898 | 5 | 11 | 0.42% | 1.80E-02 |  | nitrogen compound catabolism | | GO:0044270 | 3, 4 | 18 | | 0.77% | | 4.28E-03 | |
| regulation of cell activation | GO:0050865 | 3, 4 | 14 | 0.53% | 8.25E-03 |  | regulation of biosynthesis | | GO:0009889 | 3, 4 | 27 | | 1.15% | | 4.02E-02 | |
| regulation of cell adhesion | GO:0030155 | 3, 4 | 12 | 0.46% | 1.21E-02 |  | response to toxin | | GO:0009636 | 3 | 6 | | 0.26% | | 1.66E-02 | |
| regulation of cell migration | GO:0030334 | 4, 5 | 11 | 0.42% | 5.63E-03 |  | wound healing | | GO:0042060 | 4 | 20 | | 0.85% | | 4.89E-02 | |
| regulation of cell motility | GO:0051270 | 3, 4 | 11 | 0.42% | 5.63E-03 |  |  | |  |  |  | |  | |  | |
| regulation of cell shape | GO:0008360 | 5 | 10 | 0.38% | 3.88E-02 |  |  | |  |  |  | |  | |  | |
| regulation of development | GO:0050793 | 2, 3 | 23 | 0.88% | 1.49E-03 |  |  | |  |  |  | |  | |  | |
| regulation of locomotion | GO:0040012 | 2, 3 | 11 | 0.42% | 5.63E-03 |  |  | |  |  |  | |  | |  | |
| regulation of metabolism | GO:0019222 | 2, 3 | 395 | 15.05% | 1.68E-02 |  |  | |  |  |  | |  | |  | |
| response to biotic stimulus | GO:0009607 | 2 | 224 | 8.54% | 1.00E-03 |  |  | |  |  |  | |  | |  | |
| sequestering of calcium ion | GO:0051208 | 3 | 4 | 0.15% | 3.88E-02 |  |  | |  |  |  | |  | |  | |
| sequestering of metal ion | GO:0051238 | 2, 3 | 5 | 0.19% | 1.64E-02 |  |  | |  |  |  | |  | |  | |
| striated muscle development | GO:0007519 | >5 | 11 | 0.42% | 3.14E-02 |  |  | |  |  |  | |  | |  | |
