# Supplementary material for: Improved Elucidation of Biological Processes Linked to Diabetic Nephropathy by Single Probe-Based Microarray Data Analysis
Source: PLoS One. 2008 Aug 13;3(8):e2937. doi: 10.1371/journal.pone.0002937 (PMC2493035; doi:10.1371/journal.pone.0002937)
Supplement: Table S4 — Gene list of Wnt receptor signaling pathway, neurogenesis, and Wnt target genes. Genes of the selected GO categories are shown with their respective regulation indicated by either analysis method. Bold are the gene symbols of genes with the highest fold changes, which were selected for confirmatory studies. In addition, Wnt target genes are listed. Only fold changes indicated as significant by the respective method are shown. (0.34 MB DOC) [file pone.0002937.s004.doc]

**Table S4: Gene list of Wnt receptor signaling pathway, neurogenesis, and Wnt target genes**

Genes of the selected GO categories are shown with their respective regulation indicated by either analysis method. Bold are the gene symbols of genes with the highest fold changes, which were selected for confirmatory studies. In addition, Wnt target genes are listed. Only fold changes indicated as significant by the respective method are shown.

| Wnt receptor signaling pathway | | | |  |  |  |  |  |  |
| --- | --- | --- | --- | --- | --- | --- | --- | --- | --- |
|  |  |  | | **ChipInspector (FDR 0_max)** | |  | **RMA/SAM (q < 5%)** | | |
| **Entrez Gene ID** | **Gene Symbol** | **Gene Title** | | **Transcript** | **Fold Change** |  | **Probe Set ID** | **Transcript** | **Fold change** |
| 1452 | CSNK1A1 | casein kinase 1, alpha 1 | | NM_001892 | 1.554 |  | 206562_s_at | NM_001892 | 1.322 |
|  | CSNK1A1 | casein kinase 1, alpha 1 | | NM_001025105 | 1.554 |  | 213860_x_at | AW268585 | 1.854 |
|  | CSNK1A1 | casein kinase 1, alpha 1 | |  |  |  | 213086_s_at | BF341845 | 1.635 |
|  | CSNK1A1 | casein kinase 1, alpha 1 | |  |  |  | 208865_at | BG534245 | 1.442 |
| 1456 | CSNK1G3 | casein kinase 1, gamma 3 | | NM_004384 | 1.397 |  | 220768_s_at | NM_004384 | 1.510 |
|  | CSNK1G3 | casein kinase 1, gamma 3 | | NM_001031812 | 1.397 |  |  |  |  |
| 1457 | CSNK2A1 | casein kinase 2, alpha 1 polypeptide | | NM_001895 | 1.443 |  | 206075_s_at | NM_001895 | 1.349 |
|  | CSNK2A1 | casein kinase 2, alpha 1 polypeptide | | NM_177560 | 1.443 |  |  |  |  |
|  | CSNK2A1 | casein kinase 2, alpha 1 polypeptide | | NM_177559 | 1.443 |  |  |  |  |
| 27122 | DKK3 | dickkopf homolog 3 (Xenopus laevis) | | AK092979 | 1.795 |  | 214247_s_at | AU148057 | 2.335 |
|  | DKK3 | dickkopf homolog 3 (Xenopus laevis) | | NM_015881 | 1.795 |  |  |  |  |
|  | DKK3 | dickkopf homolog 3 (Xenopus laevis) | | NM_013253 | 1.795 |  |  |  |  |
|  | DKK3 | dickkopf homolog 3 (Xenopus laevis) | | NM_001018057 | 1.795 |  |  |  |  |
|  | DKK3 | dickkopf homolog 3 (Xenopus laevis) | | AK098756 | 1.795 |  |  |  |  |
|  | DKK3 | dickkopf homolog 3 (Xenopus laevis) | | AK090952 | 1.795 |  |  |  |  |
| 8321 | FZD1 | frizzled homolog 1 (Drosophila) | | NM_003505 | 1.472 |  | 204451_at | NM_003505 | 1.704 |
| 8323 | FZD6 | frizzled homolog 6 (Drosophila) | | NM_003506 | 2.003 |  | 203987_at | NM_003506 | 3.821 |
| 25776 | PGEA1 | PKD2 interactor, golgi and endoplasmic reticulum associated 1 | | NM_015373 | 1.348 |  | 203450_at | NM_015373 | 1.109 |
|  | PGEA1 | PKD2 interactor, golgi and endoplasmic reticulum associated 1 | | NM_001002880 | 1.348 |  |  |  |  |
| 6422 | SFRP1 | secreted frizzled-related protein 1 | | NM_003012 | 1.806 |  | 202037_s_at | NM_003012 | 2.100 |
|  | SFRP1 | secreted frizzled-related protein 1 | | AK127331 | 1.806 |  | 202036_s_at | AF017987 | 1.579 |
| 7088 | TLE1 | transducin-like enhancer of split 1 (E(sp1) homolog, Drosophila) | | NM_005077 | 1.641 |  | 203221_at | AI758763 | 2.287 |
|  |  |  | |  |  |  |  |  |  |
|  |  |  | |  |  |  |  |  |  |
| 324 | APC | adenomatosis polyposis coli | | NM_000038 | 1.480 |  |  |  |  |
| 1500 | CTNND1 | catenin (cadherin-associated protein), delta 1 | | NM_001331 | 1.500 |  |  |  |  |
| 51339 | **DACT1** | dapper, antagonist of beta-catenin, homolog 1 (Xenopus laevis) | | NM_016651 | **1.815** |  |  |  |  |
| 1856 | DVL2 | dishevelled, dsh homolog 2 (Drosophila) | | NM_004422 | 1.412 |  |  |  |  |
|  | DVL2 | dishevelled, dsh homolog 2 (Drosophila) | | AK026019 | 1.412 |  |  |  |  |
| 10023 | FRAT1 | frequently rearranged in advanced T-cell lymphomas | | NM_005479 | 1.416 |  |  |  |  |
| 23401 | FRAT2 | frequently rearranged in advanced T-cell lymphomas 2 | | NM_012083 | 1.426 |  |  |  |  |
| 2487 | FRZB | frizzled-related protein | | AK130009 | 1.575 |  |  |  |  |
|  | FRZB | frizzled-related protein | | NM_001463 | 1.575 |  |  |  |  |
| 2535 | FZD2 | frizzled homolog 2 (Drosophila) | | NM_001466 | 1.454 |  |  |  |  |
| 8324 | FZD7 | frizzled homolog 7 (Drosophila) | | NM_003507 | 1.656 |  |  |  |  |
| 5518 | PPP2R1A | protein phosphatase 2 (formerly 2A), regulatory subunit A (PR 65), alpha isoform | | AK130000 | 1.465 |  |  |  |  |
|  | PPP2R1A | protein phosphatase 2 (formerly 2A), regulatory subunit A (PR 65), alpha isoform | | AK090488 | 1.465 |  |  |  |  |
|  | PPP2R1A | protein phosphatase 2 (formerly 2A), regulatory subunit A (PR 65), alpha isoform | | AK091387 | 1.465 |  |  |  |  |
|  | PPP2R1A | protein phosphatase 2 (formerly 2A), regulatory subunit A (PR 65), alpha isoform | | NM_014225 | 1.465 |  |  |  |  |
| 5519 | PPP2R1B | protein phosphatase 2 (formerly 2A), regulatory subunit A (PR 65), beta isoform | | NM_002716 | 1.360 |  |  |  |  |
| 25928 | SOSTDC1 | sclerostin domain containing 1 | | AK093408 | 1.568 |  |  |  |  |
|  | SOSTDC1 | sclerostin domain containing 1 | | NM_015464 | 1.568 |  |  |  |  |
| 6932 | **TCF7** | transcription factor 7 (T-cell specific, HMG-box) | | NM_201632 | **1.657** |  |  |  |  |
|  | **TCF7** | transcription factor 7 (T-cell specific, HMG-box) | | NM_003202 | **1.657** |  |  |  |  |
|  | **TCF7** | transcription factor 7 (T-cell specific, HMG-box) | | NM_201634 | **1.657** |  |  |  |  |
|  | **TCF7** | transcription factor 7 (T-cell specific, HMG-box) | | NM_213648 | **1.657** |  |  |  |  |
|  | **TCF7** | transcription factor 7 (T-cell specific, HMG-box) | | AK131428 | **1.657** |  |  |  |  |
| 7089 | TLE2 | transducin-like enhancer of split 2 (E(sp1) homolog, Drosophila) | | NM_003260 | 1.436 |  |  |  |  |
|  | TLE2 | transducin-like enhancer of split 2 (E(sp1) homolog, Drosophila) | | AK123183 | 1.436 |  |  |  |  |
|  |  |  | |  |  |  |  |  |  |
| 56998 | CTNNBIP1 | catenin, beta interacting protein 1 | |  |  |  | 203081_at | NM_020248 | 0.805 |
| 1857 | DVL3 | dishevelled, dsh homolog 3 (Drosophila) | |  |  |  | 201908_at | NM_004423 | 1.225 |
| 26959 | HBP1 | HMG-box transcription factor 1 | |  |  |  | 209102_s_at | AF019214 | 1.158 |
| 51176 | **LEF1** | lymphoid enhancer-binding factor 1 | |  |  |  | 221558_s_at | AF288571 | **1.849** |
| 6934 | **TCF7L2** | transcription factor 7-like 2 (T-cell specific, HMG-box) | |  |  |  | 216511_s_at | AJ270770 | **1.305** |
|  |  |  | |  |  |  |  |  |  |
|  |  |  | |  |  |  |  |  |  |
| Neurogenesis | | | |  |  |  |  |  |  |
|  | | | | **ChipInspector (FDR 0_max)** | |  | **RMA/SAM (q < 5%)** | | |
| **Entrez Gene ID** | **Gene Symbol** | | **Gene Title** | **Transcript** | **Fold Change** |  | **Probe Set ID** | **Transcript** | **Fold change** |
| 375790 | AGRIN | | agrin | AK125197 | 1.867 |  | 212285_s_at | AW008051 | 2.012 |
|  | AGRIN | | agrin | AK021586 | 1.867 |  | 217419_x_at | AK021586 | 1.726 |
|  | AGRIN | | agrin | NM_198576 | 1.867 |  |  |  |  |
|  | AGRIN | | agrin | AK128761 | 1.867 |  |  |  |  |
| 9915 | ARNT2 | | aryl-hydrocarbon receptor nuclear translocator 2 | NM_014862 | 1.622 |  | 202986_at | NM_014862 | 1.603 |
| 928 | CD9 | | CD9 molecule | NM_001769 | 1.770 |  | 201005_at | NM_001769 | 2.051 |
| 11113 | CIT | | citron (rho-interacting, serine/threonine kinase 21) | NM_007174 | 1.523 |  | 212801_at | AI861788 | 1.408 |
| 1627 | DBN1 | | drebrin 1 | NM_004395 | 1.828 |  | 202806_at | NM_004395 | 1.437 |
|  | DBN1 | | drebrin 1 | NM_080881 | 1.828 |  |  |  |  |
|  | DBN1 | | drebrin 1 | AK094125 | 1.828 |  |  |  |  |
| 7940 | LST1 | | leukocyte specific transcript 1 | NM_205838 | 1.803 |  | 215633_x_at | AV713720 | 1.475 |
|  | LST1 | | leukocyte specific transcript 1 | NM_205840 | 1.803 |  | 214181_x_at | AI735692 | 1.493 |
|  | LST1 | | leukocyte specific transcript 1 | NM_205839 | 1.780 |  |  |  |  |
|  | LST1 | | leukocyte specific transcript 1 | NM_205837 | 1.780 |  |  |  |  |
|  | LST1 | | leukocyte specific transcript 1 | NM_007161 | 1.780 |  |  |  |  |
| 4137 | MAPT | | microtubule-associated protein tau | NM_016841 | 0.513 |  | 203928_x_at | AI870749 | 0.477 |
|  | MAPT | | microtubule-associated protein tau | NM_016835 | 0.513 |  | 206401_s_at | J03778 | 0.415 |
|  | MAPT | | microtubule-associated protein tau | NM_016834 | 0.513 |  | 203929_s_at | AI056359 | 0.383 |
|  | MAPT | | microtubule-associated protein tau | NM_005910 | 0.513 |  |  |  |  |
|  | MAPT | | microtubule-associated protein tau | AK055986 | 0.488 |  |  |  |  |
|  | MAPT | | microtubule-associated protein tau | AK095802 | 0.513 |  |  |  |  |
| 4118 | MAL | | mal, T-cell differentiation protein | NM_002371 | 1.603 |  | 204777_s_at | NM_002371 | 1.648 |
|  | MAL | | mal, T-cell differentiation protein | NM_022440 | 1.603 |  |  |  |  |
|  | MAL | | mal, T-cell differentiation protein | NM_022439 | 1.603 |  |  |  |  |
|  | MAL | | mal, T-cell differentiation protein | NM_022438 | 1.603 |  |  |  |  |
|  | MAL | | mal, T-cell differentiation protein | AK123269 | 1.603 |  |  |  |  |
| 8829 | NRP1 | | neuropilin 1 | NM_003873 | 2.086 |  | 212298_at | BE620457 | 3.386 |
|  | NRP1 | | neuropilin 1 | NM_001024628 | 1.530 |  |  |  |  |
| 56288 | PARD3 | | par-3 partitioning defective 3 homolog (C. elegans) | AK027735 | 1.434 |  | 221526_x_at | AW452651 | 1.313 |
|  | PARD3 | | par-3 partitioning defective 3 homolog (C. elegans) | NM_019619 | 1.434 |  | 210094_s_at | AF196186 | 1.216 |
|  | PARD3 | | par-3 partitioning defective 3 homolog (C. elegans) | AK024668 | 1.434 |  |  |  |  |
|  | PARD3 | | par-3 partitioning defective 3 homolog (C. elegans) | AK025892 | 1.434 |  |  |  |  |
|  | PARD3 | | par-3 partitioning defective 3 homolog (C. elegans) | AK024668 | 0.504 |  |  |  |  |
|  | PARD3 | | par-3 partitioning defective 3 homolog (C. elegans) | NM_019619 | 0.504 |  |  |  |  |
| 5376 | PMP22 | | peripheral myelin protein 22 | NM_153322 | 1.911 |  | 210139_s_at | L03203 | 1.544 |
|  | PMP22 | | peripheral myelin protein 22 | NM_153321 | 1.911 |  |  |  |  |
|  | PMP22 | | peripheral myelin protein 22 | NM_000304 | 1.911 |  |  |  |  |
| 7869 | SEMA3B | | sema domain, immunoglobulin domain (Ig), short basic domain, secreted, (semaphorin) 3B | NM_004636 | 0.628 |  | 203071_at | NM_004636 | 0.503 |
|  | SEMA3B | | sema domain, immunoglobulin domain (Ig), short basic domain, secreted, (semaphorin) 3B | AK098329 | 0.628 |  |  |  |  |
|  | SEMA3B | | sema domain, immunoglobulin domain (Ig), short basic domain, secreted, (semaphorin) 3B | NM_001005914 | 0.628 |  |  |  |  |
|  | SEMA3B | | sema domain, immunoglobulin domain (Ig), short basic domain, secreted, (semaphorin) 3B | AK092182 | 0.628 |  |  |  |  |
| 6477 | SIAH1 | | seven in absentia homolog 1 (Drosophila) | NM_003031 | 1.383 |  | 202981_x_at | NM_003031 | 1.182 |
|  | SIAH1 | | seven in absentia homolog 1 (Drosophila) | AK094663 | 1.450 |  |  |  |  |
|  | SIAH1 | | seven in absentia homolog 1 (Drosophila) | NM_001006610 | 1.450 |  |  |  |  |
| 7070 | THY1 | | Thy-1 cell surface antigen | NM_006288 | 0.430 |  | 208851_s_at | AL161958 | 0.276 |
|  | THY1 | | Thy-1 cell surface antigen | AK094374 | 0.430 |  | 208850_s_at | AL558479 | 0.339 |
|  | THY1 | | Thy-1 cell surface antigen | AK090644 | 0.533 |  | 213869_x_at | AA218868 | 0.238 |
|  | THY1 | | Thy-1 cell surface antigen | AK090996 | 0.430 |  |  |  |  |
|  | THY1 | | Thy-1 cell surface antigen | AK057865 | 0.430 |  |  |  |  |
| 7533 | YWHAH | | tyrosine 3-monooxygenase/tryptophan 5-monooxygenase activation protein, eta polypeptide | NM_003405 | 1.957 |  | 201020_at | NM_003405 | 2.890 |
|  |  | |  |  |  |  |  |  |  |
|  |  | |  |  |  |  |  |  |  |
| 348 | **APOE** | | apolipoprotein E | NM_000041 | **0.334** |  |  |  |  |
| 4897 | **NRCAM** | | neuronal cell adhesion molecule | NM_005010 | **1.592** |  |  |  |  |
|  | **NRCAM** | | neuronal cell adhesion molecule | NM_001037132 | **1.592** |  |  |  |  |
|  | **NRCAM** | | neuronal cell adhesion molecule | NM_001037133 | **1.592** |  |  |  |  |
| 5076 | PAX2 | | paired box gene 2 | NM_003988 | 1.537 |  |  |  |  |
|  | PAX2 | | paired box gene 2 | NM_003989 | 1.537 |  |  |  |  |
|  | PAX2 | | paired box gene 2 | NM_003990 | 1.537 |  |  |  |  |
|  | PAX2 | | paired box gene 2 | NM_000278 | 1.537 |  |  |  |  |
|  | PAX2 | | paired box gene 2 | NM_003987 | 1.537 |  |  |  |  |
| 6091 | ROBO1 | | roundabout, axon guidance receptor, homolog 1 (Drosophila) | NM_133631 | 1.498 |  |  |  |  |
|  | ROBO1 | | roundabout, axon guidance receptor, homolog 1 (Drosophila) | NM_002941 | 1.498 |  |  |  |  |
|  | ROBO1 | | roundabout, axon guidance receptor, homolog 1 (Drosophila) | AK025535 | 1.498 |  |  |  |  |
| 10505 | SEMA4F | | sema domain, immunoglobulin domain (Ig), transmembrane domain (TM) and short cytoplasmic domain, (semaphorin) 4F | NM_004263 | 1.394 |  |  |  |  |
|  | SEMA4F | | sema domain, immunoglobulin domain (Ig), transmembrane domain (TM) and short cytoplasmic domain, (semaphorin) 4F | AK095587 | 1.394 |  |  |  |  |
|  |  | |  |  |  |  |  |  |  |
|  |  | |  |  |  |  |  |  |  |
| 9048 | ARTN | | artemin |  |  |  | 210237_at | AF120274 | 0.753 |
| 9637 | FEZ2 | | fasciculation and elongation protein zeta 2 (zygin II) |  |  |  | 215000_s_at | AL117593 | 1.225 |
|  | FEZ2 | | fasciculation and elongation protein zeta 2 (zygin II) |  |  |  | 202305_s_at | AI685892 | 1.174 |
| 4983 | OPHN1 | | oligophrenin 1 |  |  |  | 206323_x_at | NM_002547 | 2.871 |
| 9463 | PICK1 | | protein interacting with PRKCA 1 |  |  |  | 204746_s_at | NM_012407 | 0.740 |
| 6277 | S100A6 | | S100 calcium binding protein A6 |  |  |  | 217728_at | NM_014624 | 2.490 |
| 8835 | **SOCS2** | | suppressor of cytokine signaling 2 |  |  |  | 203373_at | NM_003877 | **0.681** |
| 10417 | **SPON2** | | spondin 2, extracellular matrix protein |  |  |  | 218638_s_at | NM_012445 | **2.922** |
| 51399 | TRAPPC4 | | trafficking protein particle complex 4 |  |  |  | 217958_at | NM_016146 | 0.807 |
| 7311 | UBA52 | | ubiquitin A-52 residue ribosomal protein fusion product 1 /// ubiquitin A-52 residue ribosomal protein fusion product 1 |  |  |  | 221700_s_at | AF348700 | 1.213 |
|  |  | |  |  |  |  |  |  |  |
|  |  | |  |  |  |  |  |  |  |
| **Wnt target genes** | | |  |  |  |  |  |  |  |
|  |  | |  |  |  |  |  |  |  |
|  |  | |  | **ChipInspector (FDR 0_max)** | |  | **RMA/SAM (q < 5%)** | | |
| **Entrez Gene ID** | **Gene Symbol** | | **Gene Title** | **Transcript** | **Fold Change** |  | **Probe Set ID** | **Transcript** | **Fold change** |
| 5243 | ABCB1 | | ATP-binding cassette, sub-family B (MDR/TAP), member 1 | NM_000927 | 1.490 |  | 209993_at | AF016535 | 1.364 |
| 652 | BMP4 | | bone morphogenetic protein 4 | NM_130850 | 1.387 |  |  |  |  |
| 652 | BMP4 | | bone morphogenetic protein 4 | NM_130851 | 1.387 |  |  |  |  |
| 652 | BMP4 | | bone morphogenetic protein 4 | NM_001202 | 1.387 |  |  |  |  |
| 960 | CD44 | | CD44 molecule (Indian blood group) | AK129808 | 1.702 |  | 204489_s_at | NM_000610 | 1.743 |
| 960 | CD44 | | CD44 molecule (Indian blood group) | NM_000610 | 1.933 |  | 204490_s_at | M24915 | 1.316 |
| 960 | CD44 | | CD44 molecule (Indian blood group) | NM_001001389 | 1.933 |  | 212014_x_at | AI493245 | 1.488 |
| 960 | CD44 | | CD44 molecule (Indian blood group) | NM_001001391 | 1.933 |  | 209835_x_at | BC004372 | 1.515 |
| 960 | CD44 | | CD44 molecule (Indian blood group) | NM_001001390 | 1.933 |  | 212063_at | BE903880 | 5.610 |
| 960 | CD44 | | CD44 molecule (Indian blood group) | NM_001001392 | 1.994 |  |  |  |  |
| 960 | CD44 | | CD44 molecule (Indian blood group) | AK127622 | 2.693 |  |  |  |  |
| 999 | CDH1 | | cadherin 1, type 1, E-cadherin (epithelial) | NM_004360 | 1.970 |  | 201131_s_at | NM_004360 | 2.289 |
| 1948 | EFNB2 | | ephrin-B2 | NM_004093 | 1.543 |  | 202668_at | BF001670 | 1.743 |
| 1956 | EGFR | | epidermal growth factor receptor (erythroblastic leukemia viral (v-erb-b) oncogene homolog, avian) | NM_201284 | 1.341 |  |  |  |  |
| 1956 | EGFR | | epidermal growth factor receptor (erythroblastic leukemia viral (v-erb-b) oncogene homolog, avian) | NM_005228 | 1.387 |  |  |  |  |
| 2335 | FN1 | | fibronectin 1 | NM_212482 | 2.425 |  |  |  |  |
| 2335 | FN1 | | fibronectin 1 | NM_212478 | 2.425 |  | 210495_x_at | AF130095 | 3.263 |
| 2335 | FN1 | | fibronectin 1 | AK026737 | 2.425 |  | 216442_x_at | AK026737 | 3.288 |
| 2335 | FN1 | | fibronectin 1 | NM_212476 | 2.425 |  | 212464_s_at | X02761 | 3.740 |
| 2335 | FN1 | | fibronectin 1 | NM_212475 | 2.425 |  | 211719_x_at | BC005858 | 3.905 |
| 2335 | FN1 | | fibronectin 1 | AK094153 | 2.425 |  |  |  |  |
| 2335 | FN1 | | fibronectin 1 | NM_002026 | 2.425 |  |  |  |  |
| 2335 | FN1 | | fibronectin 1 | NM_212474 | 2.425 |  |  |  |  |
| 8324 | FZD7 | | frizzled homolog 7 (Drosophila) | NM_003507 | 1.656 |  |  |  |  |
| 2697 | GJA1 | | gap junction protein, alpha 1, 43kDa (connexin 43) | NM_000165 | 1.778 |  | 201667_at | NM_000165 | 3.080 |
| 182 | JAG1 | | jagged 1 (Alagille syndrome) | NM_000214 | 1.559 |  | 216268_s_at | U77914 | 1.379 |
| 688 | KLF5 | |  | NM_001730 | 1.616 |  |  |  |  |
| 4233 | MET | | met proto-oncogene (hepatocyte growth factor receptor) | NM_000245 | 1.829 |  | 203510_at | BG170541 | 2.242 |
| 4233 | MET | | met proto-oncogene (hepatocyte growth factor receptor) | AK025784 | 1.939 |  |  |  |  |
| **4316** | **MMP7** | | **matrix metallopeptidase 7 (matrilysin, uterine)** | **AK222980** | **7.961** |  |  |  |  |
| **4316** | **MMP7** | | **matrix metallopeptidase 7 (matrilysin, uterine)** | **NM_002423** | **7.961** |  | **204259_at** | **NM_002423** | **16.212** |
| 4897 | NRCAM | | neuronal cell adhesion molecule | NM_005010 | 1.592 |  |  |  |  |
| 4897 | NRCAM | | neuronal cell adhesion molecule | NM_001037132 | 1.592 |  |  |  |  |
| 4897 | NRCAM | | neuronal cell adhesion molecule | NM_001037133 | 1.592 |  |  |  |  |
| 7074 | TIAM1 | | T-cell lymphoma invasion and metastasis 1 | AK124647 | 1.465 |  | 213135_at | U90902 | 2.062 |
