# Supplementary material for: Improved Elucidation of Biological Processes Linked to Diabetic Nephropathy by Single Probe-Based Microarray Data Analysis
Source: PLoS One. 2008 Aug 13;3(8):e2937. doi: 10.1371/journal.pone.0002937 (PMC2493035; doi:10.1371/journal.pone.0002937)
Supplement: Table S5 — Probe set information and analysis results for the genes selected for confirmatory studies. A) Results of the analyses performed with CI and RMA are shown. Fold change and significance are given for all transcripts (for CI) or probe sets (for RMA) indicated by one of both methods to be significantly regulated. * indicates significance, n.s. = not significant, BC = expression below cut-off (see method section). SPON2 was missed by the initial version of CI (see above). B) Shown are the number of probes in a probe set, number of probes with a perfect and with a unique match, and the number of probes mapping to an exon. The information has been extracted from Eldorado (Genomatix, Germany). Accession numbers for known transcripts and probe set identification numbers are listed. SPON2 was not annotated in Eldorado at the time of analysis. The latest version gives results indicated as “corrected”. (0.15 MB DOC) [file pone.0002937.s005.doc]

**Table S5: Probe set information and analysis results for the genes selected for confirmatory studies**

**A)** Results of the analyses performed with CI and RMA are shown. Fold change and significance are given for all transcripts (for CI) or probe sets (for RMA) indicated by one of both methods to be significantly regulated.

* indicates significance, n.s. = not significant, BC = expression below cut-off (see method section). SPON2 was missed by the initial version of CI (see above).

**B)** Shown are the number of probes in a probe set, number of probes with a perfect and with a unique match, and the number of probes mapping to an exon. The information has been extracted from Eldorado (Genomatix, Germany). Accession numbers for known transcripts and probe set identification numbers are listed. SPON2 was not annotated in Eldorado at the time of analysis. The latest version gives results indicated as “corrected”.

**Table S5A**

|  |  | **Fold Change CI** | **Fold Change RMA/SAM** |
| --- | --- | --- | --- |
|  | **TCF7** |  |  |
| **Accession Number** | NM_201632 | 1.657 |  |
| **Probeset ID** | 205255_x_at, 205254_x_at |  | 1.472, BC |
|  |  |  |  |
|  | **DACT1** |  |  |
| **Accession Number** | NM_016651 | 1.815 |  |
| **Probeset ID** | 219179_at |  | BC |
|  |  |  |  |
|  | **APOE** |  |  |
| **Accession Number** | NM_000041 | 0.334 |  |
| **Probeset ID** | 203382_s_at, 212884_x_at |  | 0.314, 0.390 |
|  |  |  |  |
|  | **NRCAM** |  |  |
| **Accession Number** | NM_005010 | 1.592 |  |
| **Probeset ID** | 204105_s_at, 216959_x_at |  | 1.521, BC |
|  |  |  |  |
|  | **SOCS2** |  |  |
| **Accession Number** | transcripts listed in table 3A | n.s. |  |
| **Probeset ID** | 203373_at, 203372_s_at |  | 0.681*, BC |
|  |  |  |  |
|  | **SPON2** |  |  |
| **Accession Number** | transcripts listed in table 3A | (n.s.) |  |
| **Probeset ID** | 218638_s_at |  | 2.922* |
|  |  |  |  |
|  | **TCF7L2** |  |  |
| **Accession Number** | transcripts listed in table 3A | n.s. |  |
| **Probeset ID** | 212762_s_at, 212759_s_at, *216511_s_at* |  | BC, 0.842, *1.305** |
|  |  |  |  |
|  | **LEF1** |  |  |
| **Accession Number** | transcripts listed in table 3A | n.s. |  |
| **Probeset ID** | 221558_s_at, 221557_s_at, 210948_s_at |  | 1.849*, BC, BC |

**Table S5B**

|  |  |  |  |  |  |  |  |
| --- | --- | --- | --- | --- | --- | --- | --- |
| **Analysis Method** | **GeneSymbol** | **Accession Number** | **Probeset** | **Probes in set** | **Perfect** | **Unique** | **Mapping to exon** |
| **Found by CI** | **TCF7** | AK131428 | 205254_x_at | 11 | 10 | 10 | 9 |
|  |  |  | 205255_x_at | 11 | 11 | 7 | 7 |
|  |  | AK093683 | 205254_x_at | 11 | 10 | 10 | 8 |
|  |  | AK093530 | 205254_x_at | 11 | 10 | 10 | 8 |
|  |  |  | 205255_x_at | 11 | 11 | 7 | 6 |
|  |  | AK057580 | 205254_x_at | 11 | 10 | 10 | 8 |
|  |  |  | 205255_x_at | 11 | 11 | 7 | 6 |
|  |  | NM_003202 | 205254_x_at | 11 | 10 | 10 | 8 |
|  |  |  | 205255_x_at | 11 | 11 | 7 | 7 |
|  |  | NM_213648 | 205254_x_at | 11 | 10 | 10 | 8 |
|  |  |  | 205255_x_at | 11 | 11 | 7 | 7 |
|  |  | NM_201632 | 205254_x_at | 11 | 10 | 10 | 8 |
|  |  |  | 205255_x_at | 11 | 11 | 7 | 7 |
|  |  | NM_201633 | 205254_x_at | 11 | 10 | 10 | 0 |
|  |  |  | 205255_x_at | 11 | 11 | 7 | 0 |
|  |  | NM_201634 | 205254_x_at | 11 | 10 | 10 | 10 |
|  |  |  | 205255_x_at | 11 | 11 | 7 | 7 |
|  | **DACT1** | NM_001079520 | 219179_at | 11 | 11 | 11 | 11 |
|  |  | NM_016651 | 219179_at | 11 | 11 | 11 | 11 |
|  | **APOE** | NM_000041 | 203382_s_at | 11 | 11 | 11 | 11 |
|  |  |  | 212884_x_at | 11 | 7 | 7 | 7 |
|  | **NRCAM** | AK092330 | 204105_s_at | 11 | 11 | 11 | 5 |
|  |  |  | 216959_x_at | 11 | 10 | 10 | 9 |
|  |  | AK127035 | 204105_s_at | 11 | 11 | 11 | 0 |
|  |  |  | 216959_x_at | 11 | 10 | 10 | 3 |
|  |  | NM_005010 | 204105_s_at | 11 | 11 | 11 | 11 |
|  |  |  | 216959_x_at | 11 | 10 | 10 | 9 |
|  |  | NM_001037132 | 204105_s_at | 11 | 11 | 11 | 11 |
|  |  |  | 216959_x_at | 11 | 10 | 10 | 10 |
|  |  | NM_001037133 | 204105_s_at | 11 | 11 | 11 | 11 |
|  |  |  | 216959_x_at | 11 | 10 | 10 | 3 |
| **Found by RMA/SAM** | **SOCS2** | NM_003877 | 203373_at | 11 | 9 | 9 | 9 |
|  |  |  | 203372_s_at | 11 | 11 | 11 | 11 |
|  | **SPON2** | AK130164 | 218638_s_at | 11 | 11 | 11 | 0 / corrected: 11 |
|  |  | AK074770 | 218638_s_at | 11 | 11 | 11 | 0 / corrected: 11 |
|  |  | AK074618 | 218638_s_at | 11 | 11 | 11 | 0 / corrected: 11 |
|  |  | AK026054 | 218638_s_at | 11 | 11 | 11 | 0 / corrected: 11 |
|  |  | NM_012445 | 218638_s_at | 11 | 11 | 11 | 0 / corrected: 11 |
|  | **TCF7L2** | AK074705 | 212762_s_at | 11 | 11 | 11 | 11 |
|  |  | AK225809 | 212759_s_at | 11 | 11 | 11 | 11 |
|  |  |  | 212762_s_at | 11 | 11 | 11 | 11 |
|  |  | NM_030756 | 212759_s_at | 11 | 11 | 11 | 11 |
|  |  |  | 212762_s_at | 11 | 11 | 11 | 11 |
|  |  | *no transcript* | *216511_s_at* | *?* | *?* | *?* | *0* |
|  | **LEF1** | AK128255 | 221558_s_at | 11 | 10 | 10 | 10 |
|  |  | AK225772 | 221557_s_at | 11 | 11 | 11 | 11 |
|  |  |  | 210948_s_at | 11 | 9 | 9 | 8 |
|  |  | NM_016269 | 221557_s_at | 11 | 11 | 11 | 11 |
|  |  |  | 221558_s_at | 11 | 10 | 10 | 10 |
|  |  |  | 210948_s_at | 11 | 9 | 9 | 9 |
